# Modelling Working Memory Capacity: Is the Magical Number Four, Seven, or Does it Depend on What You Are Counting?

**DOI:** 10.5334/joc.387

**Published:** 2024-07-18

**Authors:** Sergio Morra, Paola Patella, Lorenzo Muscella

**Affiliations:** 1 Universitàdi Genova, Italy; 2 I.I.T. –Center for Neuroscience and Cognitive Systems, Italy

**Keywords:** Working memory capacity, Attention, Limited capacity, Compound Stimuli Visual Information (CSVI) task, Visual Array Task (VAT)

## Abstract

Limited attentional capacity is essential to working memory. How its limit should be assessed is a debated issue. Five experiments compare Cowan’s 4-units and Pascual-Leone’s 7-units models of limited working memory capacity, with presentation time and attention to operative schemes as potential explanations of this discrepancy. Experiments 1a–1c used the Compound Stimuli Visual Information (CSVI) task, with long versus brief presentation. Capacity was estimated with the Bose-Einstein model, assuming a different number of attending acts in each condition. Participants’ *k* estimates in both conditions were highly correlated and the means were not different, indicating that the same capacity is assessed in both conditions. Experiments 2 and 3 used the 5000-msec CSVI, and the Visual Array Task (VAT) in two conditions (5000- vs. 120-msec presentation). Capacity in the VAT was estimated with Morey’s Bayesian method. Participants’ *k* estimates in both VAT conditions were correlated, but the mean was higher with long presentation, suggesting that the long condition benefits from recoding or chunking. The *k* estimate in the CSVI correlated with the short VAT and (to a lesser degree in Exp.2) with the long VAT. The mean estimate of *k* in the CSVI was one unit more than in the short VAT. We conclude that the CSVI and the short VAT tap the same capacity, one unit of which in the short VAT is allocated to an operative scheme; we discuss how Cowan’s and Pascual-Leone’s views on limited capacity can be reconciled.

## Introduction

Limited capacity is a long-debated issue. Miller (1956) investigated a “channel capacity” that limits the amount of transmitted information, and cautiously raised the question of whether it was anything more than a coincidence that people can discriminate about 6 or 7 categories in absolute judgment and recall immediately as many chunks. In the Sixties and Seventies, popular models (e.g., Atkinson & Shiffrin, 1968; Baddeley, Thomson & Buchanan, 1975) conceived capacity limitations in terms of the size of one or more short-term stores, but subsequently the pendulum swung towards the concept that working memory capacity is essentially limited by a fixed amount of general-purpose attentional resources (e.g. Cowan, 1988; Engle, Cantor & Carullo, 1992). The idea that working memory is the activated subset of long-term memory, and that attentional resources are crucial to keep activated a limited number of chunks or representational units, is entertained by numerous current models (Anderson & Matessa, 1997; Barrouillet, Bernardin & Camos, 2004; Cowan, 2005, 2011; Engle, Kane & Tuholski, 1999; Kane et al., 2004; Oberauer, 2002; Pascual-Leone & Johnson, 2021).

Despite growing consensus on attentional limitation of working memory capacity, how to define and measure the limited attentional resource is a debated matter. A widely shared view is that attentional resources can be used to activate a finite number of chunks or representation units (e.g., Cowan, 2005, 2019; Oberauer & Hein, 2012), although some models are based on different assumptions. For instance, Baddeley (2012) attributes capacity limitations to a number of separate, specialized stores and reserves the use of attention to a central executive system; Bettencourt, Michalka, and Somers (2011) reject the concept of an item-limited capacity in favour of attentional filtering; and Ma, Husain, and Bays (2014) suggest that attention is distributed flexibly among all items, which can be represented with variable degree of precision. Other researchers proposed still different assumptions regarding the impact of attentional resources on working memory encoding (Simmering, Miller & Bohache, 2015; Sims, Jacobs & Knill, 2012). Indeed, there is converging evidence that the number of items held in working memory varies depending on several aspects of the tasks considered (Asp, Strömer & Brady, 2021; Brady, Konke & Alvarez, 2011; Simmering & Perone, 2013; Williams et al., 2022). However, it still seems possible to quantify limited capacity in a way that is theoretically meaningful and practically useful, if one uses tasks in which chunking is unlikely, carries out an accurate task analysis, and the nature of the distinct representations involved is fairly clear (Arsalidou, 2013; Cowan, 2001a; Pascual-Leone & Johnson, 2021; Vergauwe & Cowan, 2015). Thus, this article focuses on a debate *within* a framework that assumes attention allocation to a finite number of cognitive units. Is four the “magical number”, or should we still regard as plausible Miller’s (1956) hypothesis of a “channel capacity” of about seven chunks?

Cowan (2001a) reviewed extensive experimental evidence, including verbal and non-verbal materials, visual and auditory presentation modalities, spatial and temporal information, single and dual tasks, attended and unattended stimuli, and proposed that the limited focus-of-attention capacity averages about four chunks in normal adults. The title of Cowan’s (2001a) article, “The magical number four”, echoes Miller’s (1956) magical number seven, and immediately draws the reader’s attention to the main point: four chunks, not seven, is the typical limit (see also Cowan, 2015, for a historical reconstruction of the debate on Miller’s ideas). Subsequent reviews (Cowan, 2005; Cowan et al., 2008) corroborated further Cowan’s (2001a) conclusion, and other experimental evidence (Chow & Conway, 2015; Rouder et al., 2008; Shipstead et al., 2012; Unsworth & Engle, 2007) also supported the four-chunk hypothesis.

However, agreement on the four chunks limit is not general. Some researchers (e.g., Arsalidou et al., 2013; Jensen & Lisman, 1998; Kamiński, Brzezicka & Wróbel, 2011; Pascual-Leone & Johnson, 2011) claimed that this number is close to seven, consistent with Miller’s early suggestion. Reynolds et al. (2022) proposed an estimate of five in adults. Van den Berg, Awh and Ma (2014) tested different mathematical models and in the best-fitting model the estimated capacity was 6.4. In a modified Brown-Peterson paradigm, Langerock et al. (2018) reported mean spans that, when assessed in terms of total features, ranged from 6.32 to 8.02 across experiments. Wagner et al. (2021) reported a mean span of 6.53 in a novel task.

In particular, Pascual-Leone and Johnson (2011) reported young adults’ mean scores close to six in a number of “M capacity” measures; within the Theory of Constructive Operators (TCO: Pascual-Leone & Johnson, 2005, 2021), M capacity is conceived as a central attentional resource, akin to Cowan’s focus of attention. The TCO is a developmental theory, and M capacity is assumed to grow from 1 unit at about 3 years of age with an increase of 1 unit about every second year, until a capacity of possibly seven units is reached during adolescence; most of the evidence supporting the TCO comes from developmental research (for a review, see Morra et al., 2008), but some adult studies are also available, such as those reviewed by Pascual-Leone and Johnson (2011). In our lab, Morra (2001) reported a mean of 6.73 in the Figural Intersections Test, which uses visuo-spatial materials and requires finding the intersection area of a variable number of figures; Morra et al. (2013) reported means of 6.27 in the Figural Intersections Test and 6.04 in the Direction Following Task, which requires enacting verbal commands of variable complexity; and Morra (2015) reported means of 6.50 in the Figural Intersections Test, 6.53 in the Direction Following Task, and 6.21 in the Compound Stimuli Visual Information (CSVI) task. It should be noted, however, that the M capacity measures designed within the TCO framework are different from those on which Cowan estimated the size of the focus of attention. Hence, it is an open issue whether both sets of measures tap the same capacity.

Cowan’s (2001a) review was a target article open to peer commentary and Pascual-Leone (2001) commented on it arguing that four could be an underestimation, because Cowan only counted “chunks” of declarative knowledge (or “figurative schemes”, as they are called in the Piagetian and neo-Piagetian literature) disregarding the attentional resources required to activate elements of procedural knowledge (or “operative schemes”). Thus, Pascual-Leone argued that, if Cowan also counted procedural cognitive units, then he would obtain a higher capacity estimate, probably closer to seven. In turn, in his reply to commentaries, Cowan (2001b) argued that seven could be an overestimation, because the tasks on which Pascual-Leone bases his estimation involve long presentation time and fully attended stimuli, which could allow rehearsal, chunking, or other strategies to improve performance. That exchange of opinions opens the way to this study, because it indicates possible reasons for different capacity estimates obtained by different research groups, such as stimulus presentation time and allocation of attentional resources only to declarative or also procedural knowledge, implicitly calling for investigation of those possible reasons.

We intend to tackle experimentally some of the issues that underlie different estimates, and thus compare and possibly integrate the approaches taken by different research groups in studying capacity limitations. This article focuses on two tasks, the Visual Array Task (VAT: Luck & Vogel, 1997), extensively cited as evidence for a 4-unit limit (e.g., Cowan, 2001a) and the CSVI task (Pascual-Leone, 1970), often cited as evidence for M capacity growth throughout childhood and adolescence up to a capacity of 7 units (e.g., Pascual-Leone & Johnson, 2005). These two tasks share some basic components and demands, i.e., they both require manual responses to simple perceptual features of visual stimuli. Stimuli are usually presented briefly in the VAT and for longer time in the CSVI, but both tasks lend themselves easily to manipulating presentation time. In studying both tasks we consider the views reported above, (a) that presentation time may crucially affect participants’ strategies and, consequently, their capacity could be overestimated unless sufficiently brief presentation is used, and (b) that capacity estimation may require to take into account, at least for some tasks, also the amount of attention allocated to specific operations or units of procedural knowledge.

### The CSVI and the Bose-Einstein distribution

The original version of the CSVI (Pascual-Leone, 1970) was designed for children of different ages and did not use computer technology; the stimuli were presented on cards and participants responded with different motor actions. More recently, however, computerized versions of the CSVI have been developed (Hitzig, 2008; see also Morra, 2015) and used with adults. In this task, a participant views stimuli with a variable number of features and must respond to each of the relevant features that are present in a stimulus.

Consistent with the neo-Piagetian assumption that *schemes* are the units of cognition, the CSVI involves a training phase in which participants learn a set of nine stimulus-response pairs, the stimulus being a single visual feature (e.g. red, square, etc.), and the response an action defined as correct for that feature (i.e., in the computerized version, presses of different keys on a special response box). In this training phase each figure has only one relevant feature, and training continues until a participant shows errorless performance over a long sequence of trials. When a participant has learned thoroughly those nine “schemes” or s-r pairs, testing starts with compound stimuli (i.e., figures with 2 to 8 relevant features); the participant is not informed of how many relevant features are present in any compound stimulus and must respond to all of the features he/she detected. Clearly, the number of correct responses to a compound stimulus is a random variable that can take all integer values up to the number of features present in that figure.

In order to relate CSVI performance to attentional capacity, Pascual-Leone made a number of assumptions. The first is that a person’s attentional energy has a limited capacity *k* (*k* being an integer), which can simultaneously activate no more than *k* schemes. The second assumption is that, if plenty of time is available for looking at the stimulus and responding, participants will attend repeatedly to the stimulus (having *k* units of capacity available on each attending act). More specifically, it was assumed that after each attending act a participant evaluates whether he/she observed the stimulus well enough, and after *k* attending acts the participant has a feeling of attentional saturation and stops exploring the figure. Thus, with long stimulus presentation and unlimited response time, a participant will attend to each compound stimulus *k* times, each time with a capacity of activating up to *k* schemes, for a total of *k*^2^ units of capacity.

The third assumption made by Pascual-Leone is that the probability distribution of the number *x* of correct responses to items with *n* relevant features is a Bose-Einstein distribution. This is widely used in physics to model the distribution of particles (bosons) across different states; it represents the probability that a variable number *x* of states are occupied, under the assumptions that a set of *r* undistinguishable particles is randomly distributed over a set of *n* distinguishable states, and that more particles can occupy the same state. Phenomena studied in different fields of science have been modelled with this distribution (Feller, 1968; see also Ijiri & Simon, 1975; Tuckwell & Koziol, 1987). Its probability mass function is:


\[
p(x) = \left.\left({\begin{array}{*{20}{c}}
n\\
{n - x}
\end{array}} \right)\left({\begin{array}{*{20}{c}}
{r - 1}\\
{x - 1}
\end{array}} \right)\right/\left({\begin{array}{*{20}{c}}
{n + r - 1}\\
r
\end{array}} \right)
\]

and, as a more intuitive metaphor, one can think of *r* undistinguishable balls thrown to a set of *n* distinguishable boxes, with the random variable *x* representing the number of boxes that turn out to be occupied by at least one ball. Referring to the CSVI, *n* is the number of (distinct) features in a compound stimulus, *r* is the number of (undistinguishable) units of attentional capacity allocated to the stimulus, which takes the value of *k*^2^ for the reasons given above, and *x* is the random variable that expresses the number of features detected and responded to. A detailed task analysis of the CSVI task was presented by Pascual-Leone (1970). Here, suffice it to say that it seems reasonable to assume that the units of attention allocated by a participant to a certain stimulus are not individually distinguishable, because the participant’s response is informative on whether a certain feature was detected or not, but it gives no clues to whether only one or more units of attention were allocated to a certain feature, and we cannot label each unit of attention and list the particular unit(s) that were allocated to each detected feature. Thus, we can regard the Bose-Einstein distribution as a conceptually plausible model for the CSVI task.

One could ask why assuming that participants perform the task this way, instead of following a straightforward strategy of scanning mentally a list of all possible features and responding to those that are present in a stimulus. Our answer is that to discover and use this strategy one needs in the first place a very large working memory capacity, able to hold the representations of all possible features. Very few adults seem to use this efficient strategy. This could cause some error variance in estimating with the Bose-Einstein distribution their capacity; however, setting a ceiling of *k* = 9 (i.e., the number of potentially present features) in individual participants’ parameter estimation can keep this source of error to a minimum.

Extensive research (Globerson, 1983; Johnson, Fabian & Pascual-Leone, 1989; Johnson, Im-Bolter & Pascual-Leone, 2003; Miller, Pascual-Leone, Campbell & Juckes, 1989; Morra, 2015; Pascual-Leone, 1970, 1978; Pascual-Leone & Johnson, 2005; Pascual-Leone & Sparkman, 1980; de Ribaupierre et al., 1990) provided evidence supporting the Bose-Einstein model for the CSVI, usually with long stimulus presentation (5 sec), assuming *k*^2^ capacity units available for each stimulus, and found values of *k* increasing with age during childhood and adolescence as specified in the TCO.

A possible problem, however, is that the stimulus presentation time could affect capacity estimation in unknown ways, for instance because long presentation might elicit using more refined encoding or maintenance strategies (Cowan, 2001b). Consequently, it seems necessary to clarify whether and in which ways presentation time affects CSVI performance, and whether capacity estimates from the CSVI are robust to manipulation of presentation time.

Following Pascual-Leone’s line of reasoning, we assume that presenting the compound stimulus for a short time, such that saccades or repeated looks are not possible, should reduce the number of correct responses, because of reduced opportunity to attend to the stimulus and thus detect its relevant features. More specifically, we assume that with brief presentation the participant cannot attend to the stimulus *k* times, but only fewer times (according to the presentation conditions). Consequently, we predict that – although the total number of correct responses will be larger with long than brief presentation – the Bose-Einstein distribution will account for performance in both conditions, only changing the number *r* of “balls” from *k*^2^ with long presentation to 2*k, k*, and 3*k*, respectively with the brief presentation conditions of three different experiments. If the CSVI provides a valid estimate of attentional capacity, then the estimated value of *k* should be similar with both brief and long presentation. These predictions are tested in Experiments 1a–1c reported below.

### The VAT and its different methods for capacity estimation

On a typical VAT trial the participant is briefly presented with an array of colored squares, followed by a blank screen and then a test array, which either is identical to the first (no-change trials), or has one square in a different color (change trials). One of the squares in the test array is probed (in change trials the probed square is the one that changed color) and the participant must decide whether that square’s color changed or not. The set size (i.e., the number of squares in an array) varies across trials from few squares to a number that is far beyond capacity. Cowan (2001b) proposed a simple formula to estimate capacity from this task; assuming that a participant has the capacity to keep activated the representations of *k* squares during the retention interval, and the set size N is larger than *k*, then the participant will respond correctly if the probed square is one of the *k* squares maintained in working memory, and guess the correct response with probability .5 in case the probed square is one of the other N – *k*. It follows that, for N > *k*, the formula


\[k = {\mathrm{N}}({\mathrm{p(H)}} + {\mathrm{p(CR)}} - 1)\]

is a valid estimate of participants’ capacity (Cowan, 2001b; see also Rouder et al., 2011). In this formula, N is the set size, p(H) is the probability of a hit, i.e., detecting a change when it takes place, and p(CR) is the probability of a correct rejection, i.e., recognizing that the probed square was actually unchanged. This estimate of *k* must be calculated separately for each set size, and has the property of being fairly constant across set sizes, provided that N > *k*.

Studies with young adult participants often found average estimates of *k* between 3 and 4 (see Cowan, 2001a, 2001b; Rouder et al, 2011) or even lower (Xu et al., 2018). Higher *k* values, however, were also reported sometimes; namely, both Awh et al. (2007, exp. 1) and Saults and Cowan (2007, single-task condition of exp. 1) found mean *k* values of 4.9, Morey and Cowan (2005, single-task condition of exp. 1) found a mean *k* of 5.14, and Cowan et al. (2005) found mean *k* values of 5.67 in exp. 1 and 5.77 in exp. 2.

Cowan’s (2001b) formula proved to be a simple and useful way to estimate the number of items held in working memory in the VAT, but some problematic assumptions of this formula have also been noted (Morey, 2011; Morey & Morey, 2011; Rouder et al, 2011). First, in case hits are fewer than false alarms, the formula yields negative values of *k*; although this case is unlikely in most circumstances, in certain experimental designs (e.g., dual tasks) it can turn into a real problem. Second, when the set size N < *k*, error-free performance is assumed but, actually, occasional errors do occur; moreover, valid estimates of *k* can only be obtained when N is greater than the true value of *k*, but sometimes it may be difficult to decide which threshold of N should be used. Third, there could be individual differences in guessing whether a change occurred or not. Fourth, the assumption that participants always perform at their full capacity, with no lapses of attention, seems unrealistic (see also Rouder et al., 2008).

Because of these potential problems, Morey (2011; Morey & Morey, 2011) proposed an alternative method to estimate capacity from the VAT. This is based on three parameters: the size *k* of attentional capacity, the probability *z* that a participant attends to a stimulus, and the probability *g* that, in the lack of information about the probed item, a participant guesses that a change occurred. It is assumed that, in case the participant attends to an array of N items, *k* of these items will be held in working memory (or all of them will be held in working memory in case N ≤ *k*), and if the probed item is one of these *k*, a correct response will be produced; but in case the stimulus was unattended, or in case the stimulus was attended but the probed item is one of the N – *k* that exceed capacity, then the participant will guess. The three parameters *k, z*, and *g* can be estimated for the sample means, for individual participants, and for specific experimental conditions if relevant to the research design. Differently from Cowan’s (2001b) formula, which must be computed separately for each set size, with this method the parameter estimates are computed across set sizes. For details of the underlying mathematical model, see Morey (2011). The estimation process can be carried out with an open-source software, called Working Memory Modelling using Bayesian Analysis Techniques (WoMMBAT), described by Morey and Morey (2011).

We agree that WoMMBAT has several advantages with respect to more traditional methods, and therefore we use this method in Experiments 2 and 3 reported below. However, due to the more widespread use of Cowan’s (2001b) formula in the literature, we also report estimates obtained with that formula for the sake of comparison.

A basic assumption of all methods for estimating working memory capacity from the VAT is that grouping, chunking, recoding, rote or elaborative rehearsal, or other space-saving strategies are not used by the participants. Early research (Luck & Vogel, 1997) ruled out the hypothesis of verbal coding, and also found that varying presentation time of the memory array from 100 to 500 msec had no effect on performance, thus excluding that extending presentation up to half a second enables participants to use more efficient strategies. However, in view of Cowan’s (2001b) argument on a possible role of long presentation time in artifactual overestimation of capacity, we still need investigating whether a longer presentation time (such as the 5 sec presentation typically used for the CSVI) yields an inflated estimate of *k* in the VAT.

### The current study

This study is aimed at comparing and possibly integrating Cowan’s and Pascual-Leone’s views on limited capacity, by focusing on two tasks (the CSVI and the VAT) often used by either research group and considering some methodological and theoretical issues that emerged as potentially relevant in the controversies on capacity limits. In particular, we consider presentation time and attention to operative schemes as potential explanations of the discrepancy between Cowan’s and Pascual-Leone’s proposed estimates.

Our first goal is to determine whether the CSVI and the VAT tap, at least to some extent, the same capacity limitation. The correlation between capacity measures obtained from these two tasks has never been examined, because each task was used so far in separate lines of research. Our experiments 2 and 3 provide the relevant results.

Another goal of this study is examining the effect of presentation time on both tasks and its possible role in causing different capacity estimates from them. In agreement with Cowan (2001b), we expect that a 5-sec presentation time in the VAT enables participants to recode or chunk the stimulus items and use strategies, which would yield (artifactually) higher capacity estimates. We think that this prediction does not necessarily hold also for the CSVI, however. For the reasons explained above, we hypothesize that a drastic reduction of presentation time in the CSVI may not alter the capacity estimates obtained from it, provided that one sets to theoretically justified multiples of *k* (instead of *k*^2^) the *r* parameter in the Bose-Einstein distribution. This specific hypothesis is tested in Experiments 1a–1c.

The third goal of this study is examining the difference between the mean capacity estimates obtained from the CSVI and the VAT. The debate, so far, concerned results obtained in separate studies with different samples of participants. Although it is difficult to test directly the hypothesis that the VAT yields a lower capacity estimate because part of the capacity has to be allocated to operative schemes (i.e., procedural knowledge), determining the difference between the *k* estimates obtained from the two tasks can provide indirect evidence that could be used to shape more precisely an explicit model of the operative schemes that may be needed in the VAT. In this regard, our reasoning can only be indirect, because it seems difficult to make a direct comparison by devising a measure of the CSVI based on Cowan’s theory, or a VAT measure based on Pascual-Leone’s theory. To the best of our knowledge, in the literature there is no available model or task analysis of the CSVI based on Cowan’s theory, and regarding the VAT we have only Pascual-Leone’s (2001) generic remark that also operative schemes could be involved. However, we think that “translating” from one theory to the other is possible, because chunks of declarative knowledge in classical cognitive theories (including Cowan’s) correspond to Piagetian (and neo-Piagetian) figurative schemes, and Piagetian operative schemes correspond to units of procedural knowledge, although the latter are not regarded as part of the working memory content in Cowan’s model. Thus, we take an exploratory approach, comparing the established capacity estimates obtained from the “prototypical task” of each theoretical model, which have never administered so far to the same sample of participants. The results can provide information about the actual quantitative difference between these measures, and with this, at least a clue to which processes may account for that difference. Experiments 1a–1c test the impact of presentation time manipulation on capacity estimation in the CSVI. Experiment 2 turns to a direct comparison between CSVI and VAT, and in addition, tests the impact of presentation time manipulation on capacity estimation in the VAT. Experiment 3 replicates experiment 2 with some modifications for additional control.

## Experiments 1a–1c

The first three experiments tested the effect of presentation time on accuracy in the CSVI and on the capacity estimates derived from this task. Each experiment included a short and a long presentation condition.

### Method

#### Participants

*Experiment 1a:* 20 adults (13 women and 7 men) with a mean age of 27.2 years (s.d. = 7.2). Fifteen were undergraduate psychology students who participated for additional course credit; the others were students or graduates from other faculties.

The set size of 20 for this and the following two experiments was determined with a power analysis in which we set α = .05, 1 – β = .80, and *d* = .666 for a paired *t*-test. In turn, the rationale for setting *d* = .666 was that we deemed relevant a difference of at least one unit between the means of capacity measures obtained in both conditions, and previous research (e.g., Morra, 2015) indicated a standard deviation of about 1.5 for the CSVI and other measures of M capacity in this population; therefore, *d* = 1/1.5 = 0.666.

*Experiment 1b:* 20 adults (18 women and 2 men) with a mean age of 22.1 years (s.d. = 2.7), all psychology undergraduates who participated for additional course credit.

*Experiment 1c:* 20 adults (11 women and 9 men) with a mean age of 21.7 years (s.d. = 2.4), all psychology undergraduates who participated for additional course credit.

All participants had normal or corrected-to-normal vision and signed informed consent for taking part.

#### Materials and procedure: Experiment 1a

The CSVI requires responding to multiple features of a visual stimulus by pressing different keys on a special response box. The stimuli were presented on a 15-inch CRT monitor with a refresh rate of 75 ms; the participant was comfortably sitting at a viewing distance of approximately 70 cm. The relevant features were square shape, red color, large size, dashed contour, presence of a frame around the figure, presence of an X in the centre, presence of an O in the centre, presence of a bar under the figure, and purple background. The response box had 12 keys, clearly distinguishable by shape and color. Nine keys were associated each to one of the relevant features, two keys were dummy fillers, and a larger red key was an “enter” key to be pressed by the participant to signal that s/he had finished responding to a trial.

The training stimuli were 72 figures, eight of which were used to train participants on each of the nine relevant features; in each set of eight figures, the intended feature was present in four of them and absent in the other four. For each feature, one at a time, the experimenter told the participant which key was associated to it and required the participant to respond to the eight figures in the set.

The practice stimuli were 51 figures, 50 of which were divided in five blocks of 10; in each block, nine figures presented a single relevant feature (requiring the participant to press a single key followed by “enter”) and 1 presented no relevant feature (requiring to press only the “enter” key). The computer provided accuracy feedback after response to each practice stimulus. In block #1 the stimulus remained on the screen until response. Block #1 was presented again in case the participant did not produce at least 80% correct responses. In the following blocks, in case the participant had not responded within 5 s, the screen turned white but the participant was allowed to respond to the stimulus also after it disappeared. Each of blocks #2, #3, and #4 was presented again in case the participant did not produce all 10 correct responses, until perfect performance on each of these blocks. Block #5 was presented only to the participants who made one or more errors on any of the previous three blocks and was presented again in case the participant did not produce all 10 correct responses. This procedure ensured that all participants had learned thoroughly which key was associated to each feature. Finally, there was one more practice stimulus with 3 relevant features; the participant was required to press the keys associated to all relevant features of this stimulus, followed by the “enter” key. No feedback was given on this final practice stimulus.

**Figure 1:**
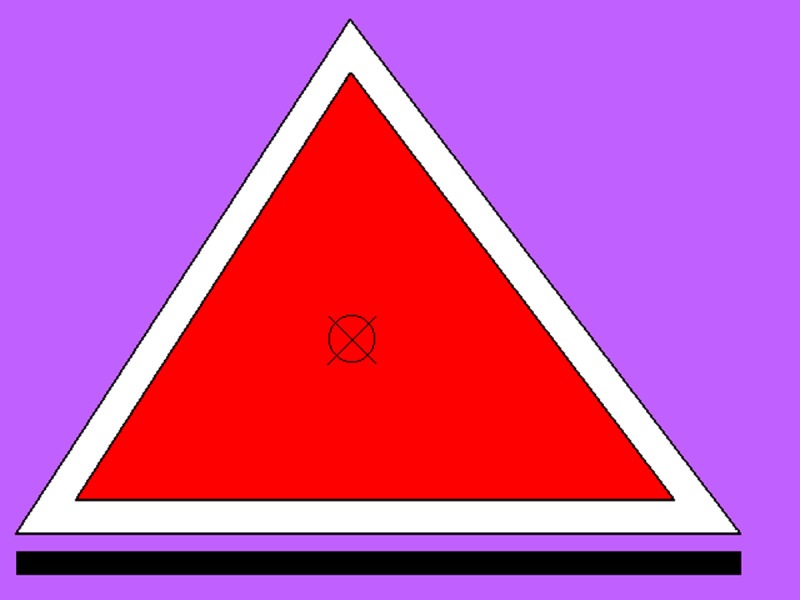
Example of a level-7 CSVI stimulus. The present features are red color, large figure, frame around the figure, X in the centre, O in the centre, bar under the figure, purple background. The absent features are square shape and dashed outline.

There were 56 test stimuli, i.e., eight trials for each level from 2 to 8, where a “level” is defined as the number of relevant features present in a stimulus. Figure 1 presents an example of a level-7 stimulus. Presentation time in the long condition was five seconds as in Pascual-Leone (1970), and in the short condition 120 msec, which prevents saccades during presentation (Spotorno, Tatler & Faure, 2013). We assume that 120 msec presentation affords only two attending acts (one while the stimulus is present and one while the iconic memory is still fully vivid).

The test stimuli were arranged in a pseudo-random fixed sequence (the same for all participants), divided into four blocks, each of which included two trials of each level. Half participants viewed each stimulus in the first and third block of for 5 s, and those in the second and fourth block for 120 ms; the presentation time of the blocks was reversed for the other half participants.^1^ A short pause was allowed after each block.

The experiment was programmed in *e-prime* (Schneider, Eschman & Zuccolotto, 2002). The session lasted approximately 50 minutes.

#### Materials and procedure: Experiments 1b and 1c

**Figure 2:**
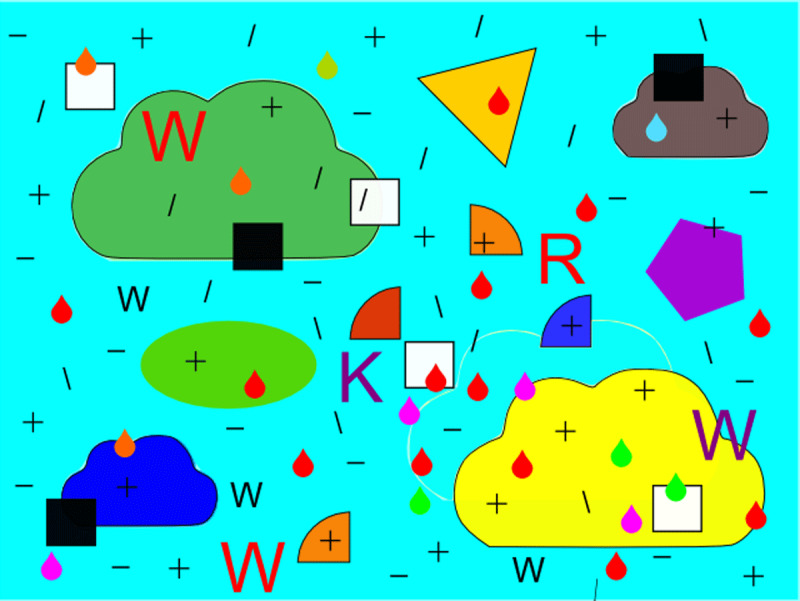
One of the six masks used in Experiments 1b and 1c.

In experiment 1b everything was identical to experiment 1a, except that in the short condition each stimulus was presented for 80 msec and followed by a mask for 500 msec. We assume that this presentation affords only one attending act. To prevent habituation to the mask, we created six different complex masks and presented one at random after each stimulus in the short condition; Figure 2 presents one of the masks.

In experiment 1c everything was identical to experiment 1b, except that in the short condition each stimulus was presented three times in a row,^2^ always for 80 msec and followed each time by a different mask for 500 msec. We assume that this presentation affords three attending acts; the participant’s responses were pooled over the three presentations.

### Results and discussion

#### Experiment 1a

Each trial was scored for the number of correct responses, i.e., correctly detected features. The participant’s total number of correct responses (maximum possible = 140) was computed in both short and long presentation conditions. The participant’s distribution of correct responses across levels was used to estimate with the Bose-Einstein model, in each presentation condition, the best fitting value of parameter *k* for his/her capacity.^3^

The participants’ total number of correct responses ranged from 72 to 126 (mean = 107.3, s.d. = 12.6) with short presentation, and from 108 to 136 (mean = 125.9, s.d. = 7.5) with long presentation. A 2 × 2 mixed ANOVA was carried out on these scores with presentation time as the within factor, and order of conditions (i.e., short-long-short-long versus long-short-long-short) as the between factor. The effect of presentation time was significant, *F*(1, 18) = 123.36, *p* <0 .001, η_p_^2^ = 0.87; nonsignificant results were obtained for order, *F*(1, 18) = 0.00, and the interaction, *F*(1, 18) = 0.06. A Bayesian ANOVA with the same design, carried out with the JASP default priors, confirmed that the best fitting model included only an effect of presentation time, BF_M_ = 5.08. This outcome was expected; this analysis was carried out merely as a manipulation check, to ensure that short presentation actually reduced participants’ ability to detect the relevant features, and that the assignment of stimulus blocks to conditions was not influential.

The actual point of interest was comparing the capacity estimates obtained in both conditions. As explained above, *k* attending acts were assumed with long presentation, but only two with short presentation. Under these assumptions, the mean estimated value of *k* was 6.85 (s.d. = 1.66) with long presentation, and 7.15 (s.d. = 1.95) with short presentation. The difference between the means was not significant, *t*(19) = –1.10, *p* > 0.28, s.e. (standard error of the difference) = 0.27, *d* = 0.16. A Bayesian paired-*t* test, with one-tailed H_1_ long > short and the prior for H_1_ set as a Cauchy distribution with scale = 0.316, supported the null hypothesis, BF_01_ = 4.08. The reason for setting the scale at 0.316 was that it is equivalent to *d* = .666, a threshold chosen for the same reasons as in the power analysis for the sample size, i.e., a mean difference of one unit divided by a standard deviation of 1.5.

Thus, although fewer relevant features were detected with short presentation, the Bose-Einstein estimates of attentional capacity in the two conditions were equivalent, provided that adequate assumptions were made on the number of attending acts in each condition. In other words, the different number of correct responses in the two presentation conditions was due to the different number of attending acts in each condition, but the participants’ capacity limit remained the same across conditions.

It is also interesting to note that neither mean value of *k* (6.85 for long and and 7.15 for short presentation) was significantly different from 7, *t*(19) = –0.40 and *t*(19) = 0.34, respectively; Bayesian analyses carried out with the JASP default priors yielded BF_01_ = 4.00 and BF_01_ = 4.08, respectively. This seems to support the view that an average capacity of about seven units is found for healthy adults in the CSVI, both with long and short presentation.

The correlation between the estimates of *k* obtained with long and short presentation was *r*(18) = .78, *p* < .001, 95% *CI* [.52, .91]; a Bayesian analyses with the JASP default priors yielded BF_10_ = 669.9. This high correlation, together with the nonsignificant difference between the means, strongly suggests that the *k* estimates obtained in both conditions measure the same construct.

#### Experiment 1b

The participants’ total number of correct responses ranged from 52 to 115 (mean = 85.1, s.d. = 17.6) with short presentation, and from 114 to 138 (mean = 125.4, s.d. = 6.5) with long presentation. In a 2 × 2 mixed ANOVA, the effect of presentation time was significant, *F*(1, 18) = 179.57, *p* < 0.001, η_p_^2^ = 0.91; nonsignificant results were obtained for order, *F*(1, 18) = 0.08, and the interaction, *F*(1, 18) = 0.49. A Bayesian ANOVA with the same design, carried out with the JASP default priors, confirmed that the best fitting model included only an effect of presentation time, BF_M_ = 6.19. Again, short presentation reduced participants’ ability to detect the relevant features, and the assignment of stimulus blocks to conditions was not influential.

The capacity estimates obtained in both conditions were compared, assuming *k* attending acts with long presentation, but only one with short presentation. Under these assumptions, the mean estimated value of *k* was 6.55 (s.d. = 1.50) with long presentation, and 6.90 (s.d. = 2.25) with short presentation. The difference between the means was not significant, *t*(19) = –1.02, *p* > 0.32, s.e. = 0.34, *d* = 0.17. A Bayesian paired-*t* test, with one-tailed H_1_ long > short and the prior for H_1_ set as a Cauchy distribution with scale = 0.316, supported the null hypothesis, BF_01_ = 3.95. Again, the capacity limit remained invariant across conditions. Moreover, neither mean value of *k* (6.55 for long and and 6.90 for short presentation) was significantly different from 7, *t*(19) = –1.34, *p* > 0.32 and *t*(19) = –0.20, respectively; Bayesian analyses carried out with the JASP default priors yielded BF_01_ = 1.98 and BF_01_ = 4.23, respectively.

The correlation between the estimates of *k* obtained with long and short presentation was *r*(18) = 0.73, *p* < 0.001, 95% *CI* [0.43, 0.89]; a Bayesian analyses with the JASP default priors yielded BF_10_ = 153.6. This high correlation, together with the nonsignificant difference between the means, strongly suggests that the *k* estimates obtained in both conditions measure the same construct.

#### Experiment 1c

The participants’ total number of correct responses ranged from 87 to 130 (mean = 111.2, s.d. = 12.4) with short presentation, and from 106 to 137 (mean = 123.3, s.d. = 7.0) with long presentation. In a 2 × 2 mixed ANOVA, the effect of presentation time was significant, *F*(1, 18) = 25.89, *p* < 0.001, η_p_^2^ = 0.59; nonsignificant results were obtained for order, *F*(1, 18) = 0.76, and the interaction, *F*(1, 18) = 1.14. A Bayesian ANOVA with the same design, carried out with the JASP default priors, confirmed that the best fitting model included only an effect of presentation time, BF_M_ = 4.79. Again, short presentation reduced participants’ ability to detect the relevant features, and the assignment of stimulus blocks to conditions was not influential.

The capacity estimates obtained in both conditions were compared, assuming *k* attending acts with long presentation, but only three with triple short presentation. Under these assumptions, the mean estimated value of *k* was 6.25 (s.d. = 1.59) with long presentation, and 6.65 (s.d. = 2.39) with short presentation. The difference between these means was not significant, *t*(19) = –0.90, *p* > 0.37, s.e. = 0.44, *d* = 0.19. A Bayesian paired-*t* test, with one-tailed H_1_ long > short and the prior for H_1_ set as a Cauchy distribution with scale = 0.316, supported the null hypothesis, BF_01_ = 3.76. Again, the participants’ capacity limit remained the same across conditions. In this sample, the mean value of *k* with short presentation (6.65) not was significantly different from 7, *t*(19) = –0.66, *p* > 0.51, with BF_01_ = 3.55 in a Bayesian analysis. However, the mean *k* with long presentation (6.25) was significantly less than 7, *t*(19) = –2.12, *p* < 0.05, with BF_10_ = 1.44 in a Bayesian analysis.

The correlation between the estimates of *k* obtained with long and short presentation was *r*(18) = 0.57, *p* < 0.01, 95% *CI* [0.16, 0.81]; a Bayesian analyses with the JASP default priors yielded BF_10_ = 6.52. Again, a positive correlation together with a nonsignificant difference between the means indicates that the *k* estimates obtained in both conditions measure the same construct.

#### Combined experiments 1a–1c

**Figure 3:**
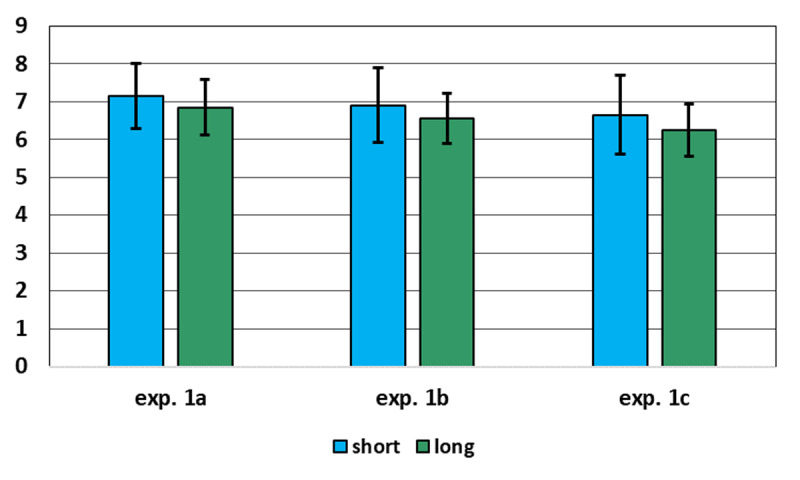
Mean and 95% CI of the k estimates in experiments 1a–1c by experiment and condition. Note that all confidence intervals overlap with one another.

In a 3 × 2 mixed ANOVA of the *k* scores, with experiments as the between factor and presentation time (short vs long) as within factor, neither effect was significant, *F*(2, 57) = 0.50, *p* > 0.61, η_p_^2^ = 0.02 for experiment, *F*(1, 57) = 2.84, *p* = 0.097, η_p_^2^ = 0.047 for presentation time, and *F*(2, 57) = 0.02, *p* > .98, η_p_^2^ = .001 for the interaction (see Figure 3). A Bayesian ANOVA with the same design, carried out with the JASP default priors, confirmed that the null model was the best fitting one, BF_M_ = 3.14. The overall means were 6.55 (s.d. = 1.58) and 6.90 (s.d. = 2.18) with long and short presentation, respectively. A Bayesian paired-*t* test, with one-tailed H_1_ long > short and the prior for H_1_ set as a Cauchy distribution with scale = 0.316, supported the null hypothesis, BF_01_ = 8.38. It can also be noted that all confidence intervals overlapped with one another. These results can be regarded as further confirmation of the robustness of the CSVI *k* scores, invariant through experiments and presentation times (provided that adequate assumptions are made regarding the attending acts occurring in each experimental condition).

It can be concluded that the mean capacity estimates of about six or seven units are not artifacts due to long presentation or to any peculiarity of the mathematical model, such as the assumption that in the long condition the participant attends *k* times to a figure.

Table 1 reports the mean number of correct responses per trial on each level of the CSVI in the long and short conditions of each experiment. It also reports the corresponding means expected from the Bose-Einstein model, under the simplifying assumption that all participants had a capacity of *k* = 7. Obviously, the means of correct responses increased with increasing levels because of the larger number of features one could respond to, and they were higher in the long than in the short condition. The interesting observation is that the ratio of correct responses in the short to the long condition tended to decrease with increasing levels. This decrease was quite dramatic in experiment 1b (where only one attending act was posited in the short condition), moderate in experiment 1a (where two attending acts were posited), and minimal in experiment 1c (where three attending acts were posited). This pattern of decreasing short-to-long observed ratios mirrored well the expected pattern predicted by the Bose-Einstein model. The correlation between the vectors of observed and predicted ratios was *r*(5) = 0.89, *p* < .01 in experiment 1a, *r*(5) = 0.98, *p* < .001 in experiment 1b, and *r*(5) = 0.84, *p* < .01 in experiment 1c; Bayesian analyses with the JASP defaults yielded BF_10_ = 16.52, BF_10_ = 433.56, and BF_10_ = 5.64, respectively in each experiment. Considering all three experiments jointly, the correlation was *r*(19) = 0.94, *p* < .001, BF_10_ = 3.82 × 10^7^. These results, consistent with the theoretical predictions, lend further support to the Bose-Einstein model.

**Table 1:** Observed and expected means of correct responses per trial, by level for short and long presentation.


	LEVEL	OBS. LONG	OBS. SHORT	OBS.SH/L RATIO	EXP. LONG	EXP. SHORT	EXP. SH/L RATIO

(a) Experiment 1a							

	2	1.99	1.89	0.95	1.96	1.87	0.95

	3	2.86	2.78	0.97	2.88	2.69	0.91

	4	3.88	3.46	0.89	3.77	3.29	0.87

	5	4.53	3.84	0.85	4.62	3.89	0.84

	6	5.39	4.44	0.82	5.44	4.42	0.81

	7	6.08	5.09	0.84	6.24	4.90	0.79

	8	6.73	5.30	0.79	6.47	4.18	0.65

(b) Experiment 1b							

	2	2.00	1.64	0.82	1.96	1.75	0.89

	3	2.93	2.31	0.79	2.88	2.33	0.81

	4	3.79	2.71	0.72	3.77	2.80	0.74

	5	4.59	3.09	0.67	4.62	3.18	0.69

	6	5.31	3.48	0.65	5.44	3.50	0.64

	7	6.11	3.91	0.64	6.24	3.77	0.60

	8	6.63	4.14	0.62	6.47	3.69	0.57

(c) Experiment 1c							

	2	1.96	1.89	0.96	1.96	1.91	0.97

	3	2.88	2.66	0.93	2.88	2.74	0.95

	4	3.71	3.44	0.93	3.77	3.50	0.93

	5	4.55	3.96	0.87	4.62	4.20	0.91

	6	5.20	4.71	0.91	5.44	4.85	0.89

	7	5.99	5.25	0.88	6.24	5.44	0.87

	8	6.54	5.88	0.90	6.47	6.00	0.93


Note: Obs. Long = Observed mean in the long condition. Obs. Short = Observed mean in the short condition. Obs. Sh/L Ratio = Ratio between observed means in the short and long conditions. Exp. Long = Expected mean from the B-E model (with *k* = 7) in the long condition. Exp. Short = Expected mean from the B-E model (with *k* = 7) in the short condition. Exp. Sh/L Ratio = Ratio between expected means in the short and long conditions.

Finally, we examined whether the Bose-Einstein distribution accounted well for the actual distribution of participants’ responses. To this effect, we considered the 240 trials performed at each level in the long condition (i.e., 4 trials per level for 60 participants) and we compared the distribution of correct responses on each level with the distribution expected from the Bose-Einstein model, and with a potentially alternative binomial model. The results provided additional support for the Bose-Einstein model. They are fully reported in the supplementary materials (see Supplementary file 1: Distributions of CSVI correct responses per level, Figure S1-1 and Tables S1-1 and S1-2). Furthermore, we examined in detail the responses to each particular feature and their temporal order (see Supplementary file 2: Responses to single CSVI features).

## Experiment 2

The second experiment tested the effect of presentation time on accuracy and capacity estimates in the VAT and compared the capacity estimates derived from VAT and CSVI. For the VAT, the same short (120 ms) and long (5 s) presentation times were used as for the CSVI in Experiment 1a; set size varied from 6 to 12. A large set size such as 12 was included to prevent ceiling performance in the long presentation condition. All CSVI stimuli were presented for 5 s, which is the standard for this task.

### Method

#### Participants

Fifty adults (37 women and 13 men) with a mean age of 22.3 years (s.d. = 3.7) took part in the experiment, including 34 psychology students who participated for additional course credit. All participants had normal or corrected-to-normal vision and signed informed consent for taking part.

Although a sample size of 20 would have been sufficient according to the power analysis reported for Experiment 1a, in this experiment we aimed at a larger sample for the purpose of obtaining more stable estimates of the capacity means, also considering the crucial importance of this experiment for the whole research project.

#### Materials and procedure

The CSVI materials were the same as in Experiments 1a–1c. Also procedure was the same, except that all test stimuli were presented for 5 s. We considered that using all 56 items in a single condition can yield more reliable estimates than using only 28, and we used long presentation because in the literature it is better established for the CSVI.

The VAT requires participants to decide, by pressing one of two keys, whether two consecutive arrangements of colored squares are identical, or the color of one of the squares was changed. The stimuli were presented on a 15-inch CRT monitor with a refresh rate of 75 ms; the participant was comfortably sitting at a viewing distance of approximately 70 cm. There were 320 test trials (80 of each set size = 6, 8, 10, and 12), in half of which the memory array was presented for 120 ms, and for 5 s in the other half; in addition, there were 16 practice items. The subsets of stimuli presented for 120 ms or 5 s were balanced over participants. Each stimulus had a grey background and included a given number (i.e., its set size) of colored squares. The squares were randomly placed within an area of 9.8° × 7.3° of visual angle at the centre of the screen; the size of each square was .75° × .75° and its color was randomly drawn with replacement from a list of 7 (white, black, red, blue, green, yellow, violet) with the only constraint that a color could not appear more than three times in a stimulus. The position of each square was randomized with the only constraint of a distance of at least 2° between any two squares. Each memory array was followed by a probe, which (with a probability of .5) was either identical to it or had a single square changed in color; one of the squares in the probe was surrounded by a circle with a diameter of 1.5°. If a square in the probe had a different color from the memory array, then the circle surrounded the changed square; otherwise, it surrounded a randomly selected square. The participant was instructed to press the keys m or z, respectively to indicate change or no change. Only accuracy was required, not speed.

**Figure 4:**
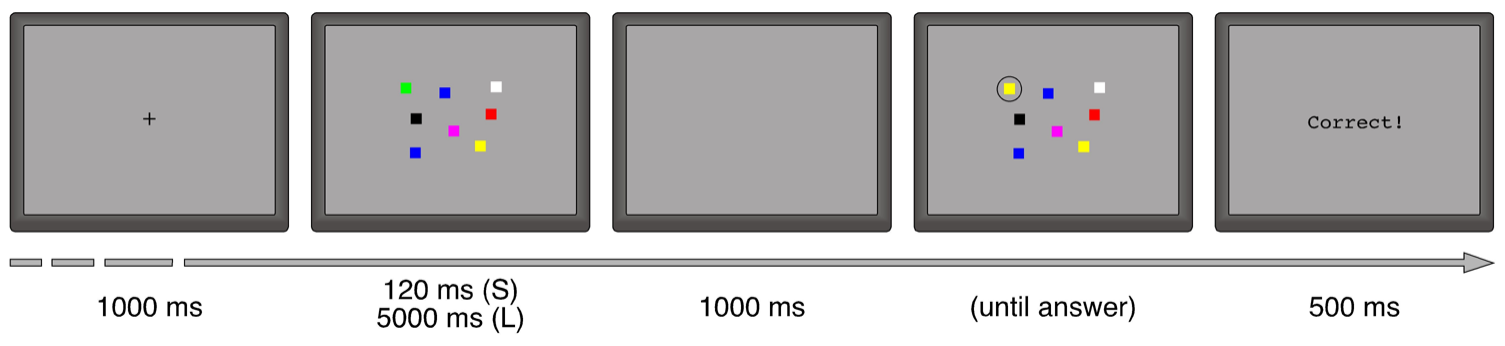
Example procedure of a VAT trial, with a stimulus of set size 8, and a color change in the probe.

A trial was started by the participant pressing a key. A fixation stimulus (a black cross in the centre of the screen) was presented for 1000 ms, followed by the memory array for 120 ms or 5 s according to the condition; then the screen remained empty for 1000 ms. At that point the probe was presented and remained visible until the participant responded. A feedback on accuracy was presented for 500 ms, then the subject was required to press a key to start the next item (see Figure 4 for an example).

The 320 test trials were divided into four blocks of 80 (20 per set size, 10 of which had a color changed in the probe). Items with different set size were randomly mixed in each block. Half participants viewed for 120 ms the memory arrays of the first and third block, and for 5 s those of the second and fourth block; the presentation time was reversed for the other half participants.

A VAT session started with two practice blocks of 8 trials each, one with 5 s and the other with 120 ms presentation. Then the four blocks of test trials followed. Pauses between blocks were allowed and the participants were also encouraged to pause within a block in case they felt tired. On average the VAT session lasted about 75 minutes, including pauses.

Both tasks were programmed in e-prime. The CSVI was administered in the first session and the VAT in the second session, with an interval of one or a few days.

### Results and discussion

Each CSVI item was scored as in Experiment 1. The participants’ total number of correct responses ranged from 209 to 272 (mean = 249.9, s.d. = 14.7) out of a maximum possible = 280, replicating well (given the doubled number of items) the results of Experiments 1a–1c with long presentation. The mean number of correct responses per trial, on levels 2–8, was 1.97, 2.91, 3.77, 4.50, 5.36, 6.00, 6.63, respectively (also similar to experiments 1a–1c). Using the Bose-Einstein model, the mean estimated value of *k* was 6.48 (s.d. = 1.54), close to the value of 6.55 found across Experiments 1a–1c.

We also examined whether the Bose-Einstein distribution accounted well for the distributions of correct responses, considering the 400 trials performed at each level. Also in this experiment, the results favored the Bose-Einstein model over the binomial model, as reported in the supplementary materials (Supplementary file 1: Distributions of CSVI correct responses per level, Figure S1-2 and Tables S1-3 and S1-4).

**Figure 5:**
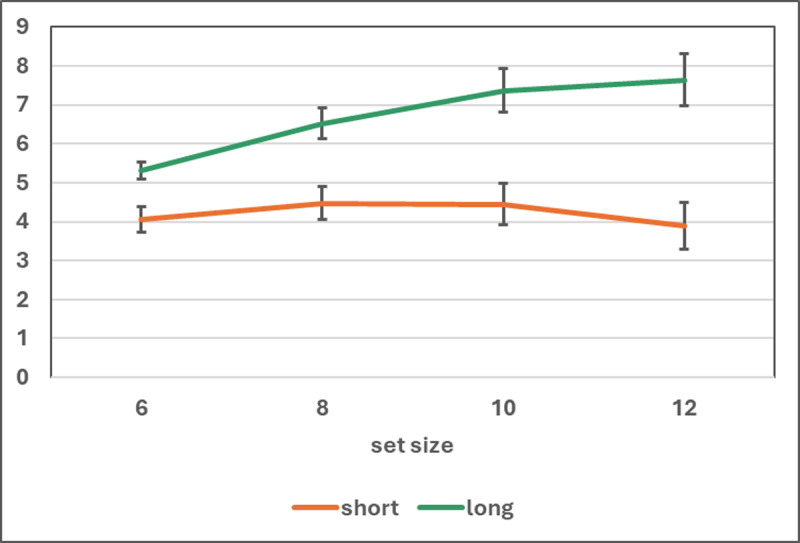
Mean and 95% CI of the *k* estimates obtained from Cowan’s (2001b) formula by presentation time and set size in Experiment 2.

The VAT data were analyzed in different ways. First, for comparison with previous studies that used Cowan’s (2001b) formula, we applied this formula to the data of each participant in each presentation condition for each set size, and the *k* values thus obtained were submitted to a 2 (presentation) x 4 (set size) repeated-measures ANOVA. There were significant effects of presentation time, *F*(1, 49) = 296.74, *p* < 0.001, η_p_^2^ = .86, *MSE* = 2.10, set size, *F*(3, 147) = 17.12, *p* < 0.001, η_p_^2^ = 0.26, *MSE* = 1.75, and the interaction, *F*(3, 147) = 21.97, *p* < 0.001, η_p_^2^ = 0.31, *MSE* = 1.33 (see Figure 5). A Bayesian ANOVA with the same design, carried out with the JASP default priors, confirmed that the best fitting model included both factors as well as their interaction, BF_M_ = 2.42 × 10^11^.

Separate analyses for short and long presentation showed that with short presentation the mean *k* was fairly constant across set sizes, *F*(3, 147) = 2.53, *p* > 0.06, η_p_^2^ = 0.05, *MSE* = 1.67. A Bayesian ANOVA with the same design confirmed that the null model was the best fitting one, BF_M_ = 1.86. The overall mean of *k*, pooled across set sizes, was 4.22 (s.d. = 1.34); this value is not significantly different from 4, *t*(49) = 1.15, n.s. (*p* > 0.25), with BF_01_ = 3.51 in a Bayesian analysis. The finding of a capacity of about 4 units, often reported from VAT studies with short presentation time and using Cowan’s formula, was replicated.

With long presentation, instead, the mean estimate of *k* varied with set size, *F*(3, 147) = 39.20, *p* < 0.001, η_p_^2^ = 0.44, *MSE* = 1.40; a Bayesian ANOVA with the same design confirmed the existence of the set size effect, BF_M_ = 4.91 × 10^15^. Contrasts between adjacent set sizes showed an increase from set size 6 to 8, *F*(1, 49) = 87.00, *p* < .001, η_p_^2^ = 0.64, *MSE* =0.83 (BF_10_ = 4.44 × 10^9^), and from set size 8 to 10, *F*(1, 49) = 17.07, *p* < 0.001, η_p_^2^ = .26, *MSE* = 2.17 (BF_10_ = 168.7); only at that point *k* leveled, the contrast between set sizes 10 and 12 yielding *F*(1, 49) = 1.00, *p* > 0.32, η_p_^2^ = .02, *MSE* = 3.75 (BF_01_ = 4.05). The mean estimate of *k*, averaging set sizes 10 and 12, was 7.51 (s.d. = 1.98).

These results suggest that in the VAT, contrary to the CSVI, the estimate of *k* is strongly affected by presentation time. This does not imply that the participants’ capacity actually varies over presentation conditions; rather, chunking and other capacity-saving strategies become possible with long presentation. During informal interviews at the end of the session, several participants reported having used strategies in the long presentation condition, such as naming the colors that were repeated in a memory array, recoding groups of squares as national flags (e.g., blue-white-red = France) or shirts of football teams, and other, more idiosyncratic strategies; they also reported that they were unable to use such strategies with short presentation. No such strategies were reported in the long presentation condition of the CSVI; a small minority of participants reported going through the list of features while selecting their responses to the CSVI, but this strategy could be used with any presentation time, because it takes place during response selection. So far, it seems that Cowan’s (2001b) suggestion that presentation time can affect capacity estimates is true of the VAT.

However, as noted in the introduction, R. Morey (2011) proposed a Bayesian method for capacity estimation that can be regarded as a methodological improvement over the classical formula. We analyzed the data separately for the short and long presentation conditions, using the WoMMBAT software (Morey & Morey, 2011), which yields parameter estimates using a Markov chain Monte Carlo method. We ran 10,000 iterations (with burn-in set at 1,000) with lower ɛ = .035 and upper ɛ = .05 for the short presentation data, and 10,000 iterations (with burn-in set at 1,000) with lower ɛ = .025 and upper ɛ = .04 for the long presentation data, thus obtaining good quality Markov chains, as indicated by the acceptance rates (.72 and .70 for short and long presentation, respectively) and by the approximately flat lines obtained for all parameters in the graphic interface, which suggest good convergence of the estimates; Geweke’s test did not reject the null hypothesis of convergence for any of the three estimated parameters in either data set.

Table 2 presents the mean and the s.d. of the individual participants’ parameter estimates obtained with this analysis. For the goals of this study it is particularly important to compare the estimates of *k* obtained in the CSVI and in the two conditions of the VAT. As reported above, participants obtained larger *k* estimates in the long than in the short VAT.

**Table 2:** Parameter estimates obtained in Experiment 2 by analyzing with WoMMBAT the VAT data separately for short and long presentation.


PRESENTATION TIME	PARAMETER	MEAN	S.D.

Short (120 ms)	*k* (capacity)	5.59	.713

	*z* (non-lapse)	.813	.084

	*g* (guessing)	.404	.138

Long (5 s)	*k* (capacity)	8.47	1.11

	*z* (non-lapse)	.892	.098

	*g* (guessing)	.502	.117


Participants obtained larger *k* estimates in the long VAT than in the CSVI, *t*(49) = 8.66, *p* < 0.001, s.e. = 0.23, *d* = 1.47. A Bayesian two-tailed paired-*t* test, with the H_1_ prior set as a Cauchy distribution with scale = 0.316, confirmed that these means were different, BF_10_ = 2.84 × 10^8^ (in this analysis we used the same scale for H_1_ as in Experiments 1a–1c; however, here we used a two-tailed test because both tasks were administered with the same long presentation time and there were no previous results or clear theoretical predictions for an expected mean score in the long VAT). Also this result is consistent with the view that a long presentation of the VAT stimuli yields an overestimation of participants’ capacity, because participants can use chunking and recoding strategies in this condition.

The mean estimate of *k* in the short VAT was somewhat higher than the value of 4, often obtained (also in this study) with Cowan’s (2001b) formula. Nevertheless, the mean estimate of *k* in the short VAT was lower than in the CSVI, *t*(49) = 4.66, *p* < 0.001, s.e. = 0.19, *d* = 0.67. A Bayesian paired-*t* test, with one-tailed H_1_ CSVI > short VAT and the prior for H_1_ set as a Cauchy distribution with scale = 0.316, supported H_1_, BF_10_ = 1420.6 (also in this analysis we used the same scale for H_1_ as in Experiments 1a–1c; here we used a one-tailed test because extensive previous results indicated higher mean capacity estimates in the CSVI than in the short VAT).

Actually, the difference between the mean *k* estimates obtained from the CSVI (6.48) and the short VAT (5.59) was close to 1 unit; testing the hypothesis that the difference between these means is equal to 1 yielded *t*(49) = –0.58, n.s. (*p* > .56), s.e. = 0.19, *d* = 0.08; a Bayesian analysis carried out with the JASP default priors yielded BF_01_ = 5.55. This would be consistent with the view that performance on the CSVI and the short VAT is limited by capacity of the same resource and in the short VAT one unit of capacity is allocated to an operative scheme.

However, before drawing strong conclusions on this point we also need to examine correlational evidence. Table 3 presents the correlations between parameter estimates obtained from the VAT and the CSVI. The correlations between different estimates of *k* are particularly relevant to the goal of this study. The *k* parameters obtained from the CSVI and the short VAT were positively correlated, *r*(48) = 0.48, *p* < .001, 95% CI [0.24, 0.67]; a Bayesian analysis with the JASP default priors yielded BF_10_ = 167.1. This, too, is consistent with the hypothesis that the same limited-capacity resource is involved in both tasks.

**Table 3:** Correlations (and 95% confidence intervals) between parameter estimates obtained from the CSVI and the VAT (analyzed with WoMMBAT) in Experiment 2.


	*K* CSVI	*K* VAT SHORT	*K* VAT LONG	*Z* VAT SHORT	*Z* VAT LONG	*G* VAT SHORT	*G* VAT LONG

*k* – CSVI	1						

*k* – VAT short	.48** *(.24, .67)*	1					

*k* – VAT long	.29* *(.01, .52)*	.57** *(.34, .73)*	1				

*z* – VAT short	.44** *(.18, .64)*	.89** *(.82, .94)*	.58** *(.36, .74)*	1			

*z* – VAT long	.54** *(.31, .71)*	.59** *(.38, .75)*	.42** *(.16, .62)*	.63** *(.43, .78)*	1		

*g* – VAT short	.01 *(–.27, .29)*	.02 *(–.26, .30)*	–.04 *(–.31, .24)*	–.04 *(–.32, .24)*	–.16 *(–.42, .12)*	1	

*g* – VAT long	–.15 *(–.41, .14)*	–.21 *(–.46, .07)*	–.03 *(–.30, .25)*	–.28 *(–.51, .002)*	–.36* *(–.58, –.10)*	.56** *(.33, .73)*	1


Note: * *p* < 0.05 two-tailed; ** *p* < 0.01 two-tailed.

The *k* estimates obtained from the CSVI and the long VAT were also positively correlated, although to a lesser extent, *r*(48) = 0.29, *p* < 0.04, 95% CI [0.01, 0.52]; in a Bayesian analysis, BF_10_ = 2.46. The correlation between CSVI and short VAT was still significant when long VAT was partialled out, *r*(47) = 0.41, *p* < 0.01, 95% CI [.14, .62], and in a Bayesian analysis, BF_10_ = 15.49, whereas the correlation between CSVI and long VAT was no longer significant when short CSVI was partialled out, *r*(47) = 0.02, *p* > 0.91, 95% CI [–0.27, 0.30), and in a Bayesian analysis, BF_01_ = 7.66. This suggests that the source of variance shared by CSVI and short VAT is shared only to a lesser extent by CSVI and long VAT. This point is notable because in this experiment both CSVI and long VAT involved 5-s stimulus presentation, but it was the *short* VAT (with only 120-ms presentation) that correlated higher with the CSVI, strengthening the conclusion that they both tap the same limited capacity.

It can also be noted in Table 3 that the estimates of *k* and *z* were positively correlated. This replicates and extends Morey’s (2011) finding of a substantial positive correlation between these two aspects of the attentional system. The estimates of *g* in both conditions correlated with each other but were uncorrelated to the other parameters.

However, a note of caution is necessary with these results. The Markov chain Monte Carlo method implemented with WoMMBAT assumes, for accurate estimation of *z*, to have at least one set size below capacity and one above capacity (Morey and Morey, 2011). Our data in the long presentation condition met this requirement, because set size ranged from 6 to 12 and the mean estimate of *k* was 8.47; in particular, with set size = 6, performance was near ceiling with an overall proportion of 0.94 accurate responses. However, our data in the short presentation condition did not meet this requirement because the mean estimate of *k*, i.e. 5.59, falls out of the 6–12 range; in particular, with set size = 6, the overall proportion correct was .84, and only 18 participants out of 50 produced at least 90% correct responses. Hence, performance on the smallest set size could not be regarded as near ceiling, which implies possible distortion or bias in the *z* estimates.

The requirement of at least one set size below capacity is only needed for estimating *z*, not *k*; however, biased estimates of *z* would in turn affect the estimates of *k* (i.e., underestimating *z* would cause an overestimate of *k*, and vice versa). A way to control for possible bias due to assumption violation is not modelling *z* but setting it at a fixed value, reasonably high to be consistent with previous research (C. C. Morey, personal communication).

Consequently, we ran WoMMBAT with the short VAT data, setting *z* at a fixed value of 0.95, and obtained a mean estimate of *k* = 4.51 (s.d. = 1.14). This mean *k* was lower than that obtained from the CSVI, *t*(49) = 9.82, *p* < 0.001, s.e. = 0.20, *d* = 1.42. A Bayesian paired-*t* test, with one-tailed H_1_ CSVI > short VAT and the prior for H_1_ set as a Cauchy distribution with scale = 0.316, supported H_1_, BF_10_ = 2.37 × 10^10^. Individual participants’ *k* estimates obtained in this way correlated positively with those obtained from the CSVI, *r*(48) = 0.47, *p* < 0.001, 95% CI [0.22, 0.66]; a Bayesian analysis with the JASP default priors yielded BF_10_ = 62.60, and this correlation remained significant when *k* estimates from the long VAT were partialled out, *r*(47) = 0.39, *p* < 0.01, 95% CI [0.12, 0.60], BF_10_ = 3.50.^4^ The difference between the mean estimate of *k* obtained from this analysis and that obtained from the CSVI was greater than 1 unit, *t*(49) = 4.83, *p* < 0.001, s.e. = 0.20, *d* = 0.68, and in a Bayesian analysis with the JASP default priors, BF_10_ = 1409.0; actually, it was close to 2 units, *t*(49) = –0.16, n.s. (*p* > 0.87), s.e. = 0.20, *d* = 0.02, and in a Bayesian analysis, BF_01_ = 6.42.

To summarize: when analyzing the short VAT data with WoMMBAT leaving *z* as a free parameter, we found mean estimates of *k* = 5.59 and *z* = 0.813; this value of *k* was lower than that obtained in the CSVI by approximately 1 unit, and the individual estimates of *k* from the CSVI and the short VAT were positively correlated. In a control analysis, with *z* fixed at .95 to control for its possible underestimation, we found a mean estimate of *k* = 4.51, and we replicated the findings that the *k* estimates from the short VAT correlated positively with those from the CSVI, and the mean *k* in the short VAT was lower than in the CSVI. These main findings, therefore, are robust against possible assumption violation in estimating *z*; the qualitative pattern of results was the same in both analyses. The only substantive difference between the results of the two analyses is that, leaving *z* as a free parameter, the short VAT mean *k* was approximately 1 unit less than the CSVI mean *k*, whereas this difference turned out to be approximately 2 units when *z* was fixed at .95. Experiment 3 was carried out to clarify whether a difference of 1 or 2 units is the most correct quantitative estimate.

## Experiment 3

The third experiment replicated the second, with some modifications intended to control for possible artifacts or confounds in Experiment 2. First, we changed the population from which the participants were drawn. As mentioned, a minority of adults could follow a more sophisticated strategy in the CSVI, which might enable them to overperform with respect to the Bose-Einstein model. This, in turn, could yield a slight overestimate of their capacity when the Bose-Einstein distribution is used to analyze their performance. This could be a source of error variance (although that strategy itself would require a large working memory, capable to hold and manage the whole list of relevant features). In this experiment we recruited adolescents (15-year-olds) as participants, assuming that they would be less likely to follow highly sophisticated strategies, although their working memory has already developed almost to reach the adult stage (Ahmed et al., 2022; Peverill et al., 2016). A second change concerned the set sizes of the VAT stimuli. Because the WoMMBAT software affords accurate estimation of all three parameters if there are at least one set size below capacity and one set size above capacity, we used set sizes 3, 6, 8, and 10, assuming that 15-year-olds would perform at ceiling on set size 3. The same hypotheses were tested as in Experiment 2, having thus eliminated two possible sources of artifacts.

Another, minor change in this experiment was dropping the informal interview on strategies at the end of each session, because all participants were enrolled in the same class and we did not want to encourage conversation among them on possible strategies in our experimental tasks.

### Method

#### Participants

A total of 24 adolescents (8 girls and 16 boys) with a mean age of 15 years and 8 months (s.d. = 3.4 months) took part in the experiment. They all attended the second class of an Italian high school and volunteered (with written parental consent) for the experiment. This was a convenience sample. A set size of 20 would have been sufficient according to the power analysis reported for Experiment 1a; the students who volunteered in that class were more than 20, so we considered that this sample was sufficiently large.

All participants had normal or corrected-to-normal vision. They were tested individually in a separate, quiet room made available by their school.

#### Materials and procedure

The materials and procedure were the same as in Experiment 2, except that the stimuli of set size = 12 were replaced by an equal number of stimuli of set size = 3.

### Results and discussion

Preliminary analyses were run to check that the changes introduced in this experiment actually had the intended consequences. The participants’ total number of correct responses in the CSVI ranged from 200 to 260 (mean = 235.0, s.d. = 17.0). The mean number of correct responses per trial, on levels 2–8, was 1.95, 2.77, 3.66, 4.37, 4.95, 5.64, 6.03, respectively (slightly lower than in the experiments with adults). Using the Bose-Einstein model, the mean estimated value of *k* was 5.29 (s.d. = 1.12), and the range of *k* was 4–7. Therefore, it seems that no participant in this experiment overperformed in the CSVI thanks to particularly sophisticated strategies. Moreover, considering the 192 trials performed at each level, also in this experiment the Bose-Einstein distribution accounted well for the distributions of correct responses, as reported in the supplementary materials (Supplementary file 1: Distributions of CSVI correct responses per level, Figure S1-3 and Tables S1-5 and S1-6).

**Figure 6:**
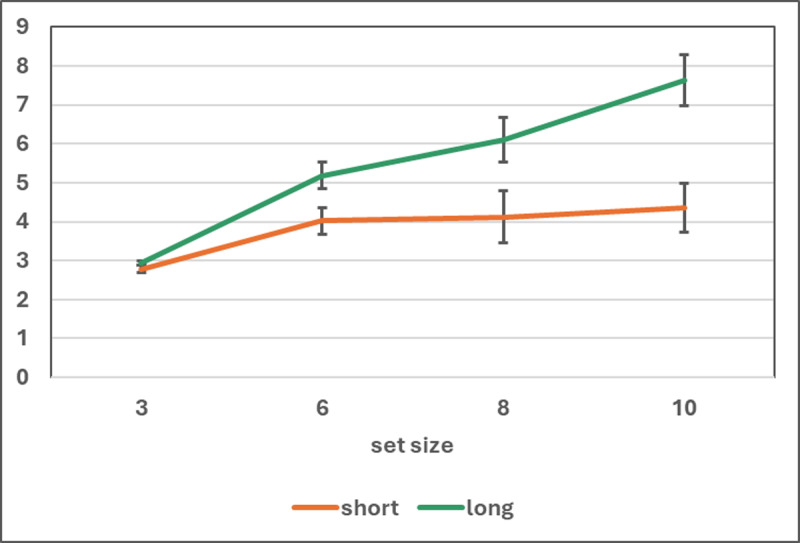
Mean and 95% CI of the *k* estimates obtained from Cowan’s (2001b) formula by presentation time and set size in Experiment 3.

The proportion of accurate responses on set size = 3 was .96 in the short and .99 in the long presentation conditions; in particular, in the short condition 23 participants out of 24 produced at least .90 correct responses, and the median was .975. Hence, performance on the smallest set size was near ceiling in both conditions (see also Figure 6), which ensures that all three WoMMBAT parameters can be modeled without biases or distortions.

The *k* values obtained from the VAT using Cowan’s (2001b) formula, presented in Figure 6, were submitted to a 2 (presentation) × 4 (set size) repeated-measures ANOVA. There were significant effects of presentation time, *F*(1, 23) = 84.84, *p* < 0.001, η_p_^2^ = 0.79, *MSE* = 1.53, set size, *F*(3, 69) = 71.19, *p* < 0.001, η_p_^2^ = 0.76, *MSE* = 1.18, and the interaction, *F*(3, 69) = 36.57, *p* < .001, η_p_^2^ = 0.61, *MSE* = 0.57. A Bayesian ANOVA with the same design, carried out with the JASP default priors, confirmed that the best fitting model included both factors as well as their interaction, BF_M_ = 6.19 × 10^13^.

Separate analyses were run for short and long presentation. With short presentation, the mean *k* estimate was not constant across set sizes, *F*(3, 69) = 12.17, *p* < 0.001, η_p_^2^ = 0.35, *MSE* = 0.99, and a Bayesian analysis yielded BF_M_ = 13945.7 for the presence of a set size effect; however, this was merely due to ceiling performance on set size = 3. When only the data from set sizes 6, 8, and 10 were analyzed, the result was nonsignificant, *F*(2, 46) = 0.65, *p* > 0.5, η_p_^2^ = 0.03, *MSE* = 1.06, with BF_M_ = 5.26 for the null model in a Bayesian analysis. The mean *k* obtained pooling these three set sizes was 4.17, which was not significantly different from 4, *t*(23) = 0.71, n.s. (*p* > 0.48), with BF_01_ = 3.71 in a Bayesian analysis.

With long presentation, instead, the mean estimate of *k* varied with set size, *F*(3, 69) = 122.53, *p* < .001, η_p_^2^ = 0.84, *MSE* = 0.76, and a Bayesian analysis yielded BF_M_ = 1.29 × 10^26^ for the presence of a set size effect; contrasts between adjacent set sizes showed increases from set size 3 to 6, *F*(1, 23) = 132.75, *p* < 0.001, η_p_^2^ = 0.88, *MSE* = 0.71 (BF_10_ = 2.28 × 10^9^), from set size 6 to 8, *F*(1, 23) = 19.47, *p* < 0.001, η_p_^2^ = 0.46, *MSE* = 1.03 (BF_10_ = 144.73), and from set size 8 to 10, *F*(1, 23) = 36.69, *p* < 0.001, η_p_^2^ = 0.62, *MSE* = 1.52 (BF_10_ = 5613.8). Replicating a finding of experiment 2, these results show that in the VAT the estimate of *k* is strongly affected by presentation time. Again, this does not imply that the participants’ actual capacity varies over presentation conditions; rather, chunking and recoding are possible with long presentation. In the case of the VAT, this finding is consistent with Cowan’s (2001b) suggestion that presentation time can affect capacity estimates.

We analyzed the VAT data using the WoMMBAT software separately for the short and long presentation conditions. We ran 20,000 iterations (with burn-in set at 2,000) with lower ɛ = 0.035 and upper ɛ = 0.05 for the short presentation data, and 20,000 iterations (with burn-in set at 2,000) with lower ɛ = 0.03 and upper ɛ = 0.045 for the long presentation data, thus obtaining good quality Markov chains, as indicated by the acceptance rates (0.65 and 0.66 for short and long presentation, respectively) and by the approximately flat lines obtained for all parameters in the graphic interface, which suggest good convergence of the estimates. In particular, Geweke’s test did not reject the null hypothesis of convergence for any of the three estimated parameters in either data set.

Table 4 presents the mean and s.d. of the individual participants’ parameter estimates obtained with this analysis. The estimates of *k* obtained in the CSVI and in the two conditions of the VAT were compared. The participants achieved larger *k* estimates in the long VAT than in the CSVI, *t*(23) = 5.95, *p* < 0.001, s.e. = 0.29, *d* = 1.08. A Bayesian two-tailed paired-*t* test, with the H_1_ prior set as a Cauchy distribution with scale = 0.316, confirmed that these means were different, BF_10_ = 2653.8 (in this analysis we used the same scale for H_1_ as in Experiments 1a–1c and a two-tailed test, as in the same analysis of Experiment 2). This is consistent with the view that a long presentation of the VAT stimuli yields an overestimation of participants’ capacity, because of chunking and recoding in this condition.

**Table 4:** Parameter estimates obtained in Experiment 3 by analyzing with WoMMBAT the VAT data separately for short and long presentation.


PRESENTATION TIME	PARAMETER	MEAN	S.D.

Short (120 ms)	*k* (capacity)	4.39	.571

	*z* (non-lapse)	.937	.030

	*g* (guessing)	.479	.133

Long (5 s)	*k* (capacity)	7.01	1.77

	*z* (non-lapse)	.964	.019

	*g* (guessing)	.540	.099


The mean *k* estimate in the short VAT was lower than in the CSVI, *t*(23) = 4.90, *p* < 0.001, s.e. = 0.18, *d* = 0.89. A Bayesian paired-*t* test, with one-tailed H_1_ CSVI > short VAT and the prior for H_1_ set as a Cauchy distribution with scale = 0.316, supported H_1_, BF_10_ = 575.0 (in this analysis we used the same scale for H_1_ as in Experiments 1a–1c and a one-tailed test, as in the same analysis of Experiment 2). So far, the results replicate those obtained in Experiment 2.

Again, the difference between the mean *k* estimates obtained from the CSVI (5.29) and the short VAT (4.39) was close to 1 unit; this replicates well the result obtained in Experiment 2 using the WoMMBAT estimates in which all three parameters were modeled. Testing the hypothesis that the difference between these means is equal to 1 yielded *t*(23) = –0.52, n.s. (*p* > 0.60), s.e. = 0.18, *d* = 0.11; a Bayesian analysis carried out with the JASP default priors yielded BF_01_ = 4.11. This would be consistent with the view that in the short VAT one unit of capacity is allocated to an operative scheme.

However, in Experiment 2 we also ran WoMMBAT with a fixed value for the parameter *z*; in this case, the mean *k* estimate obtained from the short VAT was about 2 units less than that obtained from the CSVI. In Experiment 2 that control analysis was deemed necessary, because the absence of a condition at ceiling could have introduced distortions in the parameter estimates, and therefore, although it was clear that the CSVI and short VAT estimates of *k* correlated with each other and the CSVI yielded a larger mean, Experiment 2 left undecided whether the mean difference was 1 or 2 units. In this experiment there was no risk of such confound; therefore, to clarify the size of the difference, we also tested the hypothesis that the difference between the mean *k* estimates obtained from the CSVI and the short VAT is equal to 2 units. This hypothesis, however, was rejected, *t*(23) = –5.95, *p* < 0.001, s.e. = 0.18, *d* = 1.21; a Bayesian analysis carried out with the JASP default priors yielded BF_10_ = 4414.3. Therefore, we conclude that this experiment clarified the important point that the difference between the *k* estimates obtained from the CSVI and the short VAT is approximately 1 unit, not 2.

Table 5 presents the correlations between parameter estimates obtained from the VAT and the CSVI. The *k* parameters obtained from the CSVI and the short VAT were positively correlated, *r*(22) = 0.60, *p* = 0.002, 95% CI [0.26, 0.81]; a Bayesian analysis with the JASP default priors yielded BF_10_ = 46.90. This replicates one of the main findings of Experiment 2; note that the *CI*s found in Experiments 2 and 3 for the correlation between these variables largely overlap. This positive correlation is consistent with the hypothesis that the same limited-capacity resource is involved in both tasks.

**Table 5:** Correlations (and 95% confidence intervals) between parameter estimates obtained from the CSVI and the VAT (analyzed with WoMMBAT) in Experiment 3.


	*K* CSVI	*K* VAT SHORT	*K* VAT LONG	*Z* VAT SHORT	*Z* VAT LONG	*G* VAT SHORT	*G* VAT LONG

*k* – CSVI	1						

*k* – VAT short	.60** *(.26, .81)*	1					

*k* – VAT long	.61** *(.27, .81)*	.52** *(.15, .76)*	1				

*z* – VAT short	.40 *(–.01, .69)*	.44* *(.04, .71)*	.32 *(–.09, .64)*	1			

*z* – VAT long	.14 *(–.28, .51)*	.07 *(–.34, .46)*	.07 *(–.35, .46)*	.25 *(–.17, .59)*	1		

*g* – VAT short	–.36 *(–.67, .05)*	.01 *(–.39, .41)*	–.08 *(–.47, .33)*	–.08 *(–.47, .34)*	.24 *(–.18, .59)*	1	

*g* – VAT long	.01 *(–.40, .41)*	–.03 *(–.43, .38)*	–.14 *(–.52, .28)*	–.23 *(–.58, .19)*	.32 *(–.10, .64)*	.43* *(.03, .71)*	1


Note: * *p* < 0.05, two-tailed; ** *p* < 0.01, two-tailed.

The *k* estimates obtained from the CSVI and the long VAT were also positively correlated, *r*(22) = 0.61, *p* = 0.002, 95% CI [0.27, 0.81], and in a Bayesian analysis, BF_10_ = 52.90. The correlation between CSVI and short VAT was still significant with long VAT partialled out, *r*(21) = 0.42, *p* < 0.05, 95% CI [0.01, 0.71], and in a Bayesian analysis, BF_10_ = 3.11. Also the correlation between CSVI and long VAT was significant with short VAT partialled out, *r*(21) = 0.43, *p* < 0.05, 95% CI [0.02, 0.72], and in a Bayesian analysis, BF_10_ = 3.55. In this regard, this experiment did not fully replicate the findings of the previous one. In Experiment 2, the CSVI correlated higher with the short than the long VAT; in this one, instead, both correlations were equally high, perhaps because strategy use in the long VAT was less prominent in adolescents than in university students or graduates.

It can also be noted in Table 5 that, in this experiment, some but not all estimates of *k* and *z* were positively correlated. The *z* parameter in the short condition correlated significantly (*p* < 0.04) with the *k* parameter in the short condition, and the correlation between *z* in the short condition and the *k* estimate obtained from the CSVI fell just short of significance (*p* = 0.055). This adds further evidence to the finding of positive correlations between the aspects of the attentional system indexed by *k* and *z*. The *z* estimate in the long condition, instead, did not correlate significantly with any estimate of *k*, possibly because of a ceiling effect in the *z* estimates in the long condition (see Table 4). Finally, the estimates of *g* in both conditions correlated with each other but were uncorrelated with the other parameters.

## Summary of the main findings

**Figure 7:**
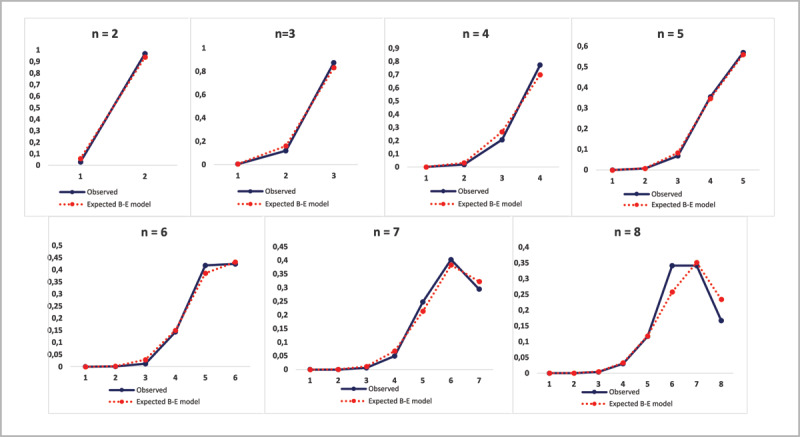
Distributions of correct responses for each CSVI stimulus level from 2 to 8 with long presentation and expected distributions from the Bose-Einstein model.

In this section we summarize and visualize the main results of this study. Figure 7 presents the distributions of the number of correct responses at each stimulus level of the CSVI with long presentation, pooled across all experiments (i.e., 832 trials in all for each level), along with the probability distributions predicted by the Bose-Einstein model. As can be seen, the observed data correspond well to the predictions, indicating that the Bose-Einstein model accounts well for the participants’ performance on the CSVI with long presentation. Detailed statistics and graphs for each experiment, tests of goodness of fit of the Bose-Einstein model and of a potentially alternative model based on the binomial distribution (which did not fit well the data) are reported in the supplementary materials (Supplementary file 1: Distributions of CSVI correct responses per level).

**Figure 8:**
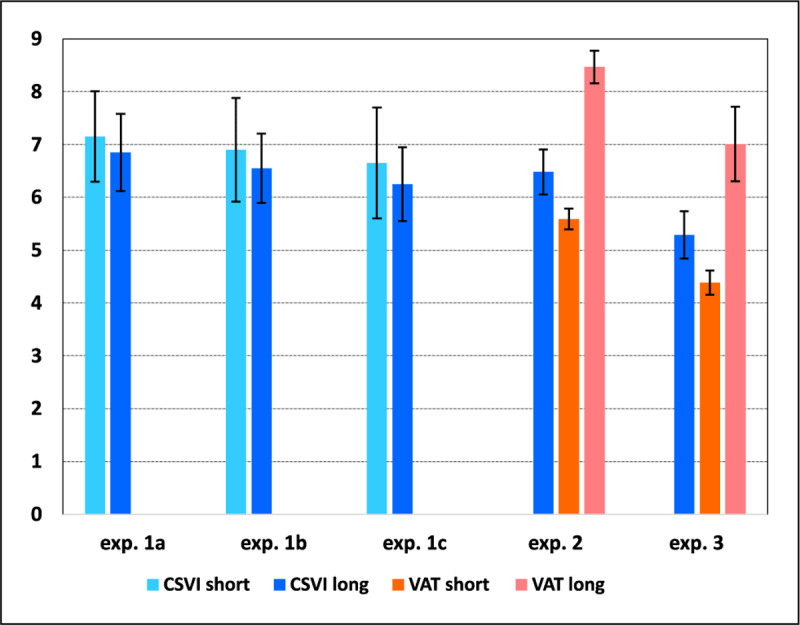
Mean and 95% CI of the *k* estimates by experiment, task, and condition.

**Figure 9:**
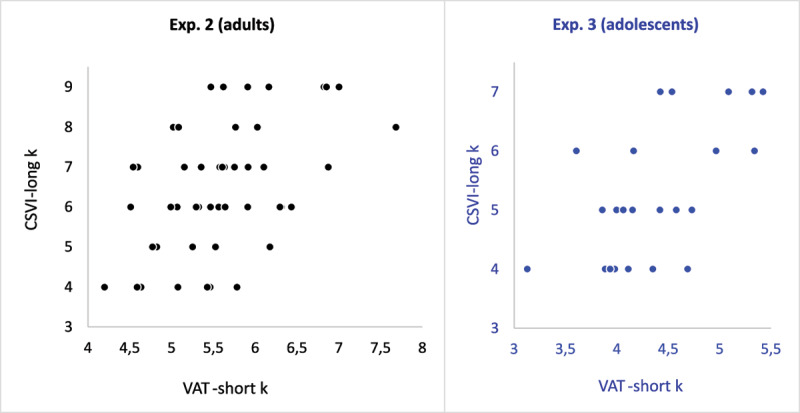
Correlation between the *k* scores in the short VAT and the long CSVI (left panel adults, right panel adolescents).

Figure 8 presents the mean *k* scores obtained with the Bose-Einstein model in the short and long conditions of the CSVI and with WoMMBAT in the short and long conditions of the VAT. One can note the stability of the CSVI across presentation times in experiments 1a–1c and 2, with mean capacity estimates ranging from 6.25 to 7.15; a lower mean score was observed only in experiment 3, with adolescent participants. In contrast, the VAT was highly sensitive to presentation time, with markedly higher scores in the long condition. Adolescents scored lower than adults also in the VAT, but the pattern of mean differences was the same in both experiments 2 and 3. The mean difference between the long CSVI and the short VAT, typically used as capacity estimates in different theoretical contexts, was approximately one unit in both experiments. Figure 9 represents graphically the correlation between the long CSVI and the short VAT.

## General Discussion

The pattern of results in these experiments seems clear. Manipulating presentation time has different effects on the CSVI and the VAT. In the VAT, a long presentation time yields higher capacity estimates, likely to be due to participants’ use of strategies. Because of this, they would not be valid as capacity estimates. This is consistent with Cowan’s (2001b) argument about possible effects of using long presentation time in tasks designed for capacity estimation. In the CSVI, instead, long presentation time yields (as expected) more correct responses but estimating capacity with the Bose-Einstein probability distribution yields equivalent capacity estimates with long and short presentation, provided that one makes appropriate assumptions on the number of “attending acts” in each condition. Moreover, the high correlations between observed and expected ratios of correct responses in the short to the long condition, and the distributions of correct responses analyzed in detail in the Supplementary File 1, provide strong support for the Bose-Einstein model of the CSVI. Therefore, the capacity estimates obtained from the CSVI are not artifacts due to long presentation time. Also the high correlations between the CSVI estimates of *k* with long and short presentation, found in experiments 1a–1c, support reliability of the capacity estimates obtained from the CSVI with either presentation time.

The pattern of correlations found in experiment 2 is important because the VAT (with 120 msec presentation) and the CSVI (with 5 sec presentation) correlated fairly well, suggesting that they tap, at least to some extent, the same capacity. One might object that the CSVI and the VAT do not share only attentional or working memory demands, but also other, more basic cognitive processes (e.g., color perception), which might account for the correlation to some extent. However, in both the VAT and the CSVI the difficulty of items is closely related to the amount of information presented (i.e., set size in the VAT, level in the CSVI), which entails that capacity limits are likely to be the main source of individual differences in each task. It seems unplausible that individual differences in color perception or other basic processes might account for a large portion of the correlation between these tasks. The correlation between CSVI and long VAT was significant but lower and did not resist partialling out the short VAT. This is also consistent with the view that the CSVI and the short VAT yield valid capacity measures, but the long VAT does not.

In experiment 3, however, this pattern of correlations was only partly replicated, because the CSVI correlated equally highly with the short and long VAT. This discrepancy, of course, could be due to statistical error in the partial correlations (a type-II error in experiment 2 or a type-I error in experiment 3); only future replication studies can tell whether this discrepancy between these two experiments is reliable. If it is, a possible explanation is that performance on the long VAT in the younger age-group depends more substantially on capacity (although recoding and chunking cause higher levels of performance than on the short VAT), whereas in adults individual differences in the efficiency of strategies are a more important determinant of performance. However, the most important correlational finding, i.e., the positive correlation between capacity estimates derived from the CSVI and the short VAT, was consistent and robust in both experiments. Moreover, the size of the correlation between CSVI and short VAT (0.48 in experiment 2 and 0.60 in experiment 3) seems no lower than the correlations between other working memory capacity measures often reported in the literature (e.g., Cowan et al., 2005).

Another notable result of experiment 2 is that the *z* parameters, both in the short and the long VAT, correlated positively with the capacity measures (both from the VAT and the CSVI). This replicates and extends a finding by Morey (2011). If *k* estimates are measures of the scope of attention, and we assume that *z* estimates are related to control of attention in the VAT, this finding would be consistent with reports of a positive correlation between scope and control of attention (Cowan et al., 2006; Unsworth et al., 2021). In experiment 3 the finding of positive correlations between the *z* parameter and the capacity measures was replicated only for the short VAT; however, in the long condition there was a clear ceiling effect on the *z* parameter, which presumably prevented correlations from emerging.

In both experiments 2 and 3, a significant difference was found between the *k* estimates derived from the CSVI and the short VAT. As noted in the introduction, we did not have a strong theoretical expectation of how large this difference would be, but our experiments intended to explore this point. Experiment 2 did not clarify whether the *k* estimate from the short VAT was 1 unit less than that from the CSVI (based on the WoMMBAT estimates with free *z* parameters) or 2 units less than that from the CSVI (based on WoMMBAT estimates with *z* parameters fixed at .95). Experiment 3, however, clarified this point by showing that the *k* estimates from the CSVI and the short VAT are positively correlated, and those from the CSVI are larger by 1 unit. This difference might be accounted for noting (in agreement with Pascual-Leone, 2001) that capacity estimates based on the VAT consider only declarative knowledge (figurative schemes), but some capacity is allocated also to procedural knowledge (operative schemes). Following this line of reasoning, the capacity allocated to procedural knowledge in the VAT would be equivalent to one unit (one operative scheme). This theoretical interpretation will be considered further below.

One might object that we used VAT arrays in which the same color could be used for more than one square. Many classical VAT studies did so; however, Morey et al. (2015; Morey, 2019) reported higher capacity estimates when a color was repeated. In those experiments, presentation time was relatively long (1200 msec), consistently with our finding that long VAT presentation enables strategy use – and grouping same-color elements would be one of them. In experiments with brief presentation (200 msec: Peterson & Berryhill, 2013), instead, perceptual grouping was found only in very facilitating conditions (circular arrangement of no more than six squares and same color in adjacent positions) not used in our experiments. Therefore, we believe that with our brief presentation of 120 msec and arrays of numerous, randomly sparse squares, the presence of repeated colors could have little effect on capacity estimates. However, this aspect could be examined in greater detail in future research; in particular, it can be hypothesized that the VAT capacity estimates show more stability across presentation times when color replication is not allowed.

### Representations and resources

The debate on working memory capacity in change-detection tasks became soon intertwined with a debate on representation. Are whole objects or their distinct features the basic units? Luck and Vogel (1997) found that participants maintained in working memory an equal number of one-featured or four-featured objects, and therefore claimed that representations of whole objects are the basic units of visual working memory. However, subsequent research demonstrated that, in the case of complex objects in which multiple features are bound together, both objects and features have a role, the relevant representations being structured hierarchically (Brady et al., 2011; Cowan, Blume & Saults, 2013; Langerock et al., 2018). This seems consistent with both Cowan’s and Pascual-Leone’s theories, which assume that long-term memory representations (Cowan, 1988) or schemes (Pascual-Leone & Goodman, 1979) have a multi-level, hierarchical structure and any level of representation can be attended to. It is also consistent with neuropsychological evidence that both objects and their properties are represented in the brain during maintenance in working memory (e.g., Luria et al., 2010; Thyer et al., 2022; Wilson et al., 2012). However, the materials we used for the VAT had only one relevant feature (color); therefore, our capacity estimations do not need modelling both levels of a hierarchical representational structure of the objects in an array, and we can safely assume that each square counts as a single unit in the focus of attention. Similarly, the CSVI concerned the features of a single object; therefore, each relevant feature can be regarded as a distinct unit, with no need of modelling a hierarchical structure.

#### On domain-general limited capacity

In the literature, the VAT data are sometimes interpreted as indicators of limited capacity of a “visuo-spatial working memory”. However, in the context of Cowan’s theory, they are regarded as indicators of the limited capacity of a domain-general focus of attention. Similarly, the CSVI was proposed by Pascual-Leone (1970) as a measure of domain-general attentional capacity. We suggest that our finding that capacity estimates from the CSVI and the short VAT are correlated reflects their use of domain-general resources, such as those posited in both Cowan’s and Pascual-Leone’s theories. This is consistent with structural equations modelling studies showing that general-purpose attentional resources are involved in visuo-spatial working memory tasks (e.g., Kane et al., 2004), and with research showing a mutual interference between visuo-spatial and verbal working memory tasks (Morey & Cowan, 2005; Uittenhove et al., 2019; Vergauwe, Barrouillet & Camos, 2010; Vergauwe et al., 2012; Vergauwe et al., 2022) and between visual and auditory stimuli (Maezawa & Kawahara, 2021; Saults & Cowan, 2007) – a mutual interference that can only be accounted for by positing a role of domain-general resources. It is also consistent with psychophysiological evidence that the attentional networks in the brain provide a system of pointers to sustain activation in the appropriate posterior processing systems, and working memory capacity is determined by the number of such pointers that the interparietal sulcus and/or the prefrontal cortex can hold (Cowan, 2019; Ruchkin et al., 2003; Thyer et al., 2022). Furthermore, Morey (2018) persuasively argued against the very existence of a specialized visuospatial short-term store. Therefore, we believe that a domain-general interpretation of the capacity limits found in the CSVI and the short VAT is appropriate.

#### Attention to objects and features

The debate on objects and features is relevant to the current study because the VAT requires change detection in an array of spatially separate objects, whereas the CSVI requires detection of a variable number of features in a complex object. Studies of brain activity during the interstimulus interval in the VAT suggest that maintenance of objects and features could involve attention in different ways. In particular, Wilson et al. (2012) claim that the amplitude of contralateral delayed activity is a function of both the number of object representations to be maintained and the number of features bound within an object; however, the activity related to features seems to be more posterior than that related to separate objects. Wilson et al. (2012) also suggest that one system holds discrete, coarse-resolution placeholders and another system encodes within-object features (see also Luria et al., 2010; Xu & Chun, 2006).

Findings that both objects and features draw on general attentional resources, but involve different representational systems, invite some speculation on how general resources are allocated to specific systems. Pascual-Leone (1970) suggested that, in the CSVI, attentional units allocated to features are stochastically distributed like bosons, i.e., undistinguishable particles that can occupy a same position. Keeping with this analogy from physics, we suggest that attentional units allocated to coarse representations of objects in space (as required in the VAT) might behave like fermions, i.e., undistinguishable particles that cannot occupy the same position.

Awh and Jonides (2001) suggested that the mechanisms of spatial attention are recruited in the service of a rehearsal-like function to maintain information active in working memory. We note that such a rehearsal function would be maximally efficient if *one* unit of attentional resources is allocated to *each* object/position; this is also consistent with Zhang and Luck’s (2011) finding that the cognitive system gives high priority to representing the largest possible number of separate objects. Therefore, we should assume different mathematical models for allocation of attention to features-in-objects and objects-in-space. When a task is based on a rich representation of manifold features (such as the CSVI), inefficiency and low performance might derive from allocating most of the available resources to just a part of the target features, while disregarding or not representing other features. In tasks (as the VAT) in which the position of several objects must be remembered, inefficiency might arise from occasionally not using all the available units of attention to rehearse the objects’ positions.

This in turn invites to consider possible further improvement of the methods used to assess capacity from the VAT. We believe that Cowan’s (2001b) formula provided a useful first approximation to capacity estimation, and Morey’s (2011) Bayesian method marked an important progress by assuming, among other things, that participants could have occasional lapses of attention and thus not always perform at their full capacity. However, Morey’s assumption was based on all-or-none allocation of attention, i.e., the model assumes that a participant either attends to the stimulus with his/her full capacity or does not attend to it at all. Perhaps a further refinement of Morey’s model might consider the possibility of partial use of resources. The precise form of such a refined model could be determined with further research, but one possibility is that if only single units of attention (behaving like fermions) are allocated to each object/position, and a person has *h* units available to represent the positions of objects, then on a given trial that person shall “waste”, i.e. not use, at least one “fermion” with a probability *w*, and leave unused at least two “fermions” with probability *w*^2^, … and use none of the available “fermions” (i.e., do not attend at all to the stimulus, as in the case of Morey’s “lapses of attention”) with probability *w*^h^. Assumptions of this sort would complicate Morey’s model, and future research can determine whether this is really needed; however, it might sharpen further the dynamics of attention allocation in the model.^5^

### Accounting for the mean difference between CSVI and short VAT

#### Declarative and procedural working memory

We have ruled out the hypothesis that the CSVI overestimates capacity due to long presentation time, because equivalent estimates were obtained with brief presentation in experiments 1a–1c. We turn now to the alternative account that the CSVI yields higher estimates than the VAT because of discounting the information load of procedural knowledge, i.e., the possibility that considering also resources allocated to operative schemes (procedural knowledge) in addition to figurative schemes (declarative knowledge) would bring the capacity estimates obtained from the VAT closer to those obtained from Pascual-Leone’s task. With this, we do not imply that Cowan’s account of capacity is wrong, or it overlooks something; it is a legitimate choice to model working memory considering as its basic units either chunks of declarative long-term memory or also procedural knowledge. Indeed, we think that Cowan’s and Pascual-Leone’s models can be reconciled precisely because they *both* are based on a capacity-limited, domain-general attentional resource, and according to which basic units one assumes, their estimates of capacity are *meaningfully* different. However, this distinction takes us to the issue of declarative and procedural information in working memory.

Oberauer (2009, 2010) inaugurated this debate by asking how the “storage” and the “processing” aspect of working memory relate to each other, and hypothesized that declarative and procedural working memory are different subsystems; he tentatively suggested that “declarative and procedural working memory have separate capacities, so that their contents don’t interfere with each other, but their capacities are limited by a common factor that varies across individuals, so that they are highly correlated.” (Oberauer, 2010, p.297). However, Gade et al. (2014) did find some interference between declarative and procedural working memory, leading these authors to doubt the hypothesis of separate subsystems. Even stronger evidence was reported by Barrouillet et al. (2015) and Vergauwe, Camos, and Barrouillet (2014). Their results indicate that declarative and procedural working memory draw on common resources, consistent with the assumptions of several neo-Piagetian models (e.g., Case, 1985; Pascual-Leone & Johnson, 1979, 2021).

#### Procedural information in the VAT

If we assume that attentional resources are shared by operative and figurative schemes (or procedural and declarative working memory), then we can suggest, as a possible explanation of the difference of 1 unit between the capacity estimates obtained in the CSVI and the short VAT, that the VAT may require activating one unit of procedural knowledge (i.e., one operative scheme) in addition to a number of declarative units (figurative schemes) that can be estimated with Morey and Morey’s (2011) method. This suggestion begs the question of what this operative scheme is. We speculate that the “rehearsal-like function” proposed by Awh and Jonides (2001) for spatial working memory could be the operative scheme involved in performing the VAT. In the verbal domain, there is evidence (Guttentag, 1984; Morra, 2000) that (at least in children) rehearsal is an operation that demands a small amount of attentional effort. By analogy, it seems reasonable to suggest that also in the spatial domain a rehearsal function is a useful operation that can be counted as a single unit (i.e., a structured procedure that does not require assembling more basic procedural components), and requires attention to be carried out – more precisely, one unit of attention, because it is present in the cognitive system as a unitary procedural representation.

### Limitations and prospects

A possible limitation of our study is not having considered those versions of the VAT in which a continuous dimension (e.g., orientation, distance, or shade of color) of an object must be recalled (e.g., Bays, Catalao & Husain, 2009). Those experiments were often cited in support of theories (e.g., Ma et al., 2014) claiming that attention is distributed flexibly among all items, with no limit on their number. Hardman, Vergauwe & Ricker (2017), however, demonstrated that no more than one color is represented in working memory in a continuous fashion, the others being represented categorically. Future research could extend our approach to estimation of capacity for continuous and categorical representations.

An important limitation is having used only two tasks. We suggest extending this line of research in the future, also taking into consideration verbal tasks and the specific problems they pose for capacity estimation (e.g., Camos, Mora & Oberauer, 2011; Cowan et al., 2012).

Moreover, future research could explore further the methodological differences between the two tasks used in this study, for instance, by creating “hybrid” versions of the tasks. The VAT could be modified by presenting a single array and requiring the participants to detect all colors present in that array; one could test whether the Bose-Einstein distribution fits the data in that condition. In principle, one could also modify the CSVI by requiring participants to detect changes between two consecutive compound stimuli; however, this procedure might be less reliable, because capacity estimates in the CSVI are robust to violations of the assumption of feature equiprobability for the reasons given in the supplementary materials (Supplementary file 2: Responses to single CSVI features), but a change detection procedure might be too sensitive to salience differences among features.

Another limitation is having compared only two theories of capacity of the attentional resources used to activate a small number of chunks or schemes. Other working memory theories also posit limited attentional capacity, framed in terms of attentional control (Unsworth & Engle, 2007), time-based resource sharing (Barrouillet, Portrat & Camos, 2011), or interference removal (Oberauer et al., 2012). Future research could extend comparison to these theoretical frameworks, considering also tasks and experimental paradigms (e.g., complex span) used by these researchers.

Finally, it would be interesting to extend this research to developmental trends. Cowan (2016) and Pascual-Leone and Johnson (2005) agree that maturation has a primary role in the development of capacity, and Pascual-Leone (1970) posited a developmental pattern with an increase of one unit approximately every second year. It would be important to establish, if possible, a similar pattern in the context of Cowan’s theory, and compare the developmental patterns of the capacity measures used most often in either theoretical framework.

## Conclusions

Despite limitations, we hope that the present comparison of the four-chunk and the seven-scheme hypotheses is a useful step towards integration of two different views of limited working memory capacity. Having found a positive correlation and a systematic difference of one unit between the VAT and the CSVI we can conclude that both views are not so incompatible with each other as it might appear on first impression, but they can be integrated by specifying, for each task on which each view was grounded, the respective roles of declarative and procedural information and the different mechanisms of attention allocation. By doing this, one can better understand what is actually measured by each task.

This study suggests that Cowan’s and Pascual-Leone’s models of limited capacity can be reconciled. The CSVI and the VAT seem to tap the same limited attentional capacity. In the VAT a brief presentation time is essential for valid capacity estimation (as suggested by Cowan, 2001b). In the CSVI, however, varying presentation time does not affect the capacity estimates, provided that one makes appropriate assumptions on the number of “attending acts” that take place in different presentation conditions. The capacity estimates obtained from the VAT and the CSVI are correlated, but their means differ, and this difference could be accounted (as suggested by Pascual-Leone, 2001) by attention allocated to operative schemes, which is not captured by methods that provide an estimation only of the attentional capacity allocated to figurative schemes (declarative representations). More precisely, a difference of one unit between the VAT and the CSVI estimates could be accounted for by the participants’ use of one operative scheme in the VAT – for instance, the “rehearsal-like function” proposed by Awh and Jonides (2001).

## Data Accessibility Statement

The data of the experiments are publicly available in the Open Science Framework at osf.io/ras3y DOI 10.17605/OSF.IO/RAS3Y with the following reference: Muscella, Lorenzo. (2024, April 24). VAT – CSVI.

## Additional Files

The additional files for this article can be found as follows:

10.5334/joc.387.s1 Supplementary file 1. Distributions of CSVI correct responses per level.

10.5334/joc.387.s2 Supplementary file 2. Responses to single CSVI features.

## Footnotes

A pilot experiment ensured that the difficulty of the odd and even blocks was not different.

The authors are grateful to Nelson Cowan for suggesting this manipulation.

More specifically, a participant’s vector of the mean numbers of correct responses on levels 2 to 8 was compared with all vectors of expected means generated by the Bose-Einstein model for each value of *k* from 2 to 9; the *k* value that yielded the smallest chi-square was selected as the best fitting.

The correlation between the estimates of *k* from the short VAT, obtained setting *z* as a free parameter in WoMMBAT or fixing its value at 0.95, was *r*(49) = 0.98, 95%CI [0.96, 0.99], i.e. was nearly perfect, although the mean value of *k* decreased by about one unit when *z* was fixed.

In the CSVI, modelling partial use of the available resources is unessential, because it would have little consequence on behaviour whether, say, 3 attentional units are allocated to a certain feature, or only 1 attentional unit is allocated to that feature and 2 units are unused. In the VAT, instead, our assumption that one attentional unit is allocated to each separate square entails that modeling the distribution of how many of the available units are actually used by a participant over all trials is relevant.
