# Supplementary material for: Modelling Working Memory Capacity: Is the Magical Number Four, Seven, or Does it Depend on What You Are Counting?
Source: J Cogn. 2024 Jul 18;7(1):60. doi: 10.5334/joc.387 (PMC11259112; doi:10.5334/joc.387)
Supplement: Supplementary file 1. — Distributions of CSVI correct responses per level. [file joc-7-1-387-s1.pdf]

S. Morra, P. Patella, L. Muscella - *Modelling Working Memory Capacity: Is the Magical Number Four, Seven, or Does it Depend on what you are Counting?*

Supplementary file 1

Distributions of CSVI correct responses per level

Experiments 1a-1c

Because in the long condition (5000 ms presentation) each participant in experiments 1a-1c performed 4 trials per level, with 60 participants in all we can draw on 240 data points for each of the distributions from level 2 to 8.

We classified the participants according to their *k*-value estimated in the long presentation condition. Bose-Einstein (B-E) distributions were generated for all values of n from 2 to 8 and all values of *k* from 4 to 9 (i.e., for the complete range of values found in the participants). Then, expected distributions for the total sample were obtained, for each n, as a weighted average of the distributions for each *k* value, with weights proportional to the number of participants who obtained that *k* value. The goodness of fit of these distributions to the data was assessed by means of chi-square tests. For these tests, whenever the expected frequency of a value of *x* (i.e., of a certain number of correct responses) was < 1, both the expected and the observed frequencies for that *x* were collapsed with the following value of *x*.

The observed distributions and the distributions predicted from the B-E model are shown in Figure S1-1. Table S1-1 presents the goodness of fit of the B-E model (i.e., the chi-squares for the comparisons between observed and expected distributions, along with their probabilities) for each of these distributions.

Figure S1-1: Distributions of correct responses for each stimulus level from 2 to 8 (each from 240 data points) and expected distributions from the Bose-Einstein and the Binomial models.

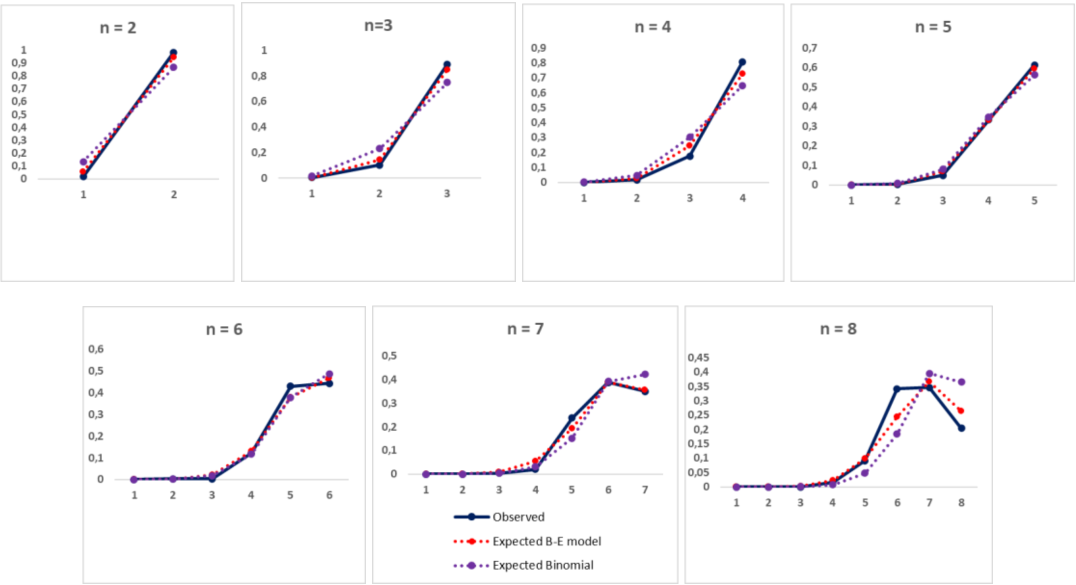

As an alternative model, to be contrasted with the B-E, we devised a binomial model. In this binomial model we assumed that, when a stimulus was presented, at least one feature would be detected, and the other features would be detected with a certain probability p, to be estimated from the data. The estimated value of p was .866. This alternative model has some face plausibility, because it makes simple assumptions on dichotomous events (each feature can either be detected or not), but it does not assume limited attentional capacity or indistinguishable units of attentional resources. The goodness of fit of the distributions predicted by this binomial model was tested in the same way as for those predicted by the B-E model.

Table S1-1: Goodness of fit of the Bose-Einstein model to the observed distributions.

n	χ^2	d.f.	*p*	
2	6.47	1	.011	*
3	3.39	2	.183	
4	8.34	2	.015	*
5	1.66	3	.647	
6	4.47	3	.215	
7	7.65	4	.105	
8	13.84	4	.008	**

*Note: * p<.05, ** p<.01 for the discrepancy between an observed and an expected distribution*

The distributions predicted from the binomial model are also shown in Figure S1-1. Table S1-2 presents the goodness of fit of the B-E model for each of these distributions.

Both Figure S1-1 and Table S1-1 indicate that the B-E distributions fit the data reasonably well. Four out of seven distributions showed a good fit ($p > .1$) and in two other cases (n=2 and n=4) the discrepancy between expected and observed distributions, although significant, was actually very small. Only for n=8 there is some notable difference between observed and observed distributions, the observed scores being slightly lower than predicted by the model.

Table S1-2: Goodness of fit of the Binomial model to the observed distributions.

n	χ^2	d.f.	*P*	
2	28.50	1	9 E-8	***
3	25.93	2	2 E-6	***
4	27.25	2	1 E-6	***
5	4.62	3	.202	
6	4.32	3	.229	
7	15.31	3	.002	**
8	63.18	4	6 E-13	***

*Note: * p<.05, ** p<.01 , *** p<.001 for the discrepancy between an observed and an expected distribution*

Figure S1-1 and Table S1-2 show that the binomial model, instead, did not fit well the data. Only two of the seven distributions fit the data well, and in the other five cases the discrepancies between observed and expected distributions were much larger. Also in the case (n=8) where the fit of the B-E model was least satisfactory, still the B-E model was much closer to the observed data than the binomial model was.

One could still wonder whether the good fit of the B-E model to the data might be an artifact, due to the calculation of a weighted average of six B-E distributions (for the six estimated values of *k* found in different participants). To check for this possibility we computed, in the same way as above, the goodness of fit of 42 B-E distributions (i.e., 7 values of n times 6 values of *k*), in order to detect any possible bias or interaction between *k* values and the fit of the distributions. We do not report here the details of this analysis, but we only mention that, out of 42 tests, only 4 showed a significant (*p*<.05) discrepancy between the observed and expected distributions. In particular, the participants with *k*=5 performed better than predicted on level-2 stimuli, and with smaller variance than predicted on level-7 stimuli; the participants with *k*=7 performed better than predicted on level-4 stimuli; and the participants with *k*=9 performed better than predicted on level-7 stimuli. No systematic bias or effect for different values of *k* could be detected, and therefore we can rule out the possibility that there was any artifact due to averaging B-E distributions for different groups of participants.

Experiment 2

In experiment 2, each participant performed 8 trials per level in the long condition. Thus, 50 participants provided 400 data points for each distribution from level-2 to 8.

The distributions expected from the B-E and the binomial model were computed in the same way as for experiments 1a-1c. Also in this case the participants' *k* scores ranged from 4 to 9. The estimated value of p for the binomial model was .862. The goodness of fit of the distributions was assessed with chi-square tests. Also for this experiment, whenever the expected frequency of a value of *x* was < 1, both the expected and the observed frequencies for that *x* were collapsed with the following value of *x*.

The observed distributions and the distributions predicted from the B-E and the binomial models are shown in Figure S1-2. Table S1-3 presents the chi-square values of the B-E model and Table S1-4 those of the binomial model for each of these distributions.

Figure S1-2: Distributions of correct responses for each stimulus level from 2 to 8 (each from 400 data points) in experiment 2 and expected distributions from the Bose-Einstein and the binomial models.

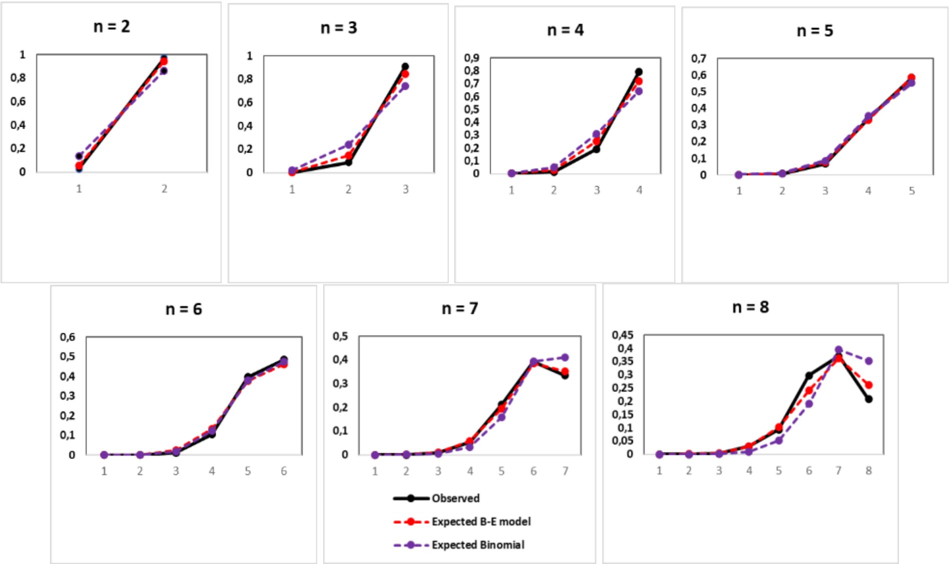

Figure S1-2 and Table S1-3 indicate that the B-E distributions slightly but significantly underestimated the participants' performance on levels 2 to 4, and fit the data well on levels 5 to 8. Fine-grained analyses of 42 B-E distributions (i.e., 7 values of n times 6 values of *k*) did not point to any bias or interaction between *k* values and the fit of the distributions. Out of 42 tests, only one showed a significant discrepancy between observed and expected distributions; in particular, the participants with *k*=5 performed better than predicted (*p*<.02) on level-3 stimuli. In contrast, as shown in Figure S1-2 and Table S1-4, the binomial model seriously underestimated performance on levels 2 to 4, fit the data well on levels 5 and 6, and seriously overestimated performance on levels 7 and 8. Overall, also in this experiment the B-E model fit the data reasonably well, and certainly better than the binomial model.

Table S1-3: Goodness of fit of the Bose-Einstein model to the observed distributions in experiment 2.

N	χ^2	d.f.	*p*	
2	5.95	1	.015	*
3	12.92	2	.002	**
4	10.83	2	.004	**
5	0.31	3	.958	
6	6.77	3	.079	
7	1.55	4	.818	
8	10.29	5	.067	

*Note: * p<.05, ** p<.01 for the discrepancy between an observed and an expected distribution*

Table S1-4: Goodness of fit of the Binomial model to the observed distributions in experiment 2.

N	χ^2	d.f.	*p*	
2	41.12	1	1 E-10	***
3	58.91	2	2 E-13	***
4	42.08	3	4 E-9	***
5	2.62	3	.454	
6	2.75	3	.432	
7	18.13	4	.002	**
8	87.01	4	6 E-18	***

*Note: * p<.05, ** p<.01 , *** p<.001 for the discrepancy between an observed and an expected distribution*

Experiment 3

In experiment 3, each participant performed 8 trials per level in the long condition. Thus, 24 participants provided 192 data points for each distribution from level-2 to 8.

The distributions expected from the B-E and the binomial model were computed in the same way as for the previous experiments. In experiment 3 the participants' *k* scores ranged from 4 to 7. The estimated value of p for the binomial model was .799. The goodness of fit of the distributions was assessed with chi-square tests. Also for this experiment, whenever the expected frequency of a value of *x* was < 1, both the expected and the observed frequencies for that *x* were collapsed with the following value of *x*.

The observed distributions and the distributions predicted from the B-E and the binomial models are shown in Figure S1-3. Table S1-5 presents the chi-square values of the B-E model and Table S1-6 those of the binomial model for each of these distributions.

Figure S1-3: Distributions of correct responses for each stimulus level from 2 to 8 (each from 192 data points) in experiment 3 and expected distributions from the Bose-Einstein and the binomial models.

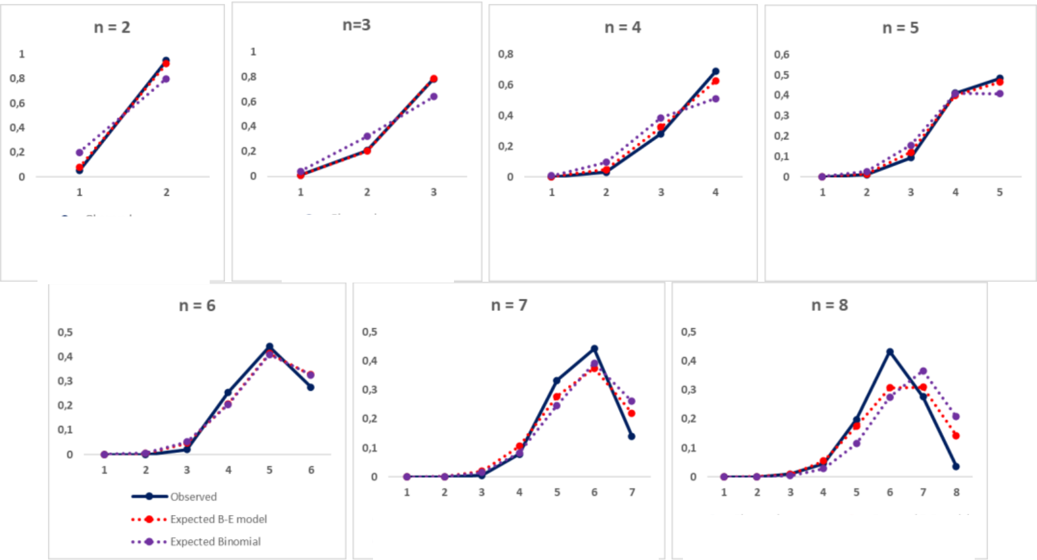

Figure S1-3 and Table S1-5 indicate that the B-E distributions fit the data well on levels 2 to 6, but overestimated the participants' performance on levels 7 and 8. Fine-grained analyses of 28 B-E distributions (i.e., 7 values of n times 4 values of *k*) did not point to any systematic bias or interaction between *k* values and the fit of the distributions. Out of 28 tests, four showed a significant discrepancy between observed and expected distributions; in particular, the participants with *k*=4 performed worse than predicted on level-6 stimuli, those with *k*=5 performed worse than predicted on level-8 stimuli, and those with *k*=7 performed better than predicted on level-4 stimuli and worse than predicted on level-8 stimuli. In contrast, as shown in Figure S1-3 and Table S1-6, the binomial model seriously underestimated performance on levels 2 to 4 and to a lesser extent on level-5, fit the data well on level-6, and seriously overestimated performance on levels 7 and 8. On levels 7 and 8, where there was a significant discrepancy between the B-E model and the data, still the B-E model performed better than the binomial. Overall, also in this experiment the B-E model fit the data reasonably well.

Table S1-5: Goodness of fit of the Bose-Einstein model to the observed distributions in experiment 3.

N	χ^2	d.f.	*p*	
2	1.71	1	.191	
3	0.05	2	.976	
4	3.65	2	.161	
5	1.53	3	.675	
6	7.53	3	.057	
7	13.75	4	.008	**
8	26.41	5	7 E-5	***

*Note: * p<.05, ** p<.01, *** p<.001 for the discrepancy between an observed and an expected distribution*

Table S1-6: Goodness of fit of the Binomial model to the observed distributions in experiment 3.

N	χ^2	d.f.	*p*	
2	26.44	1	3 E-7	***
3	17.92	2	2 E-4	***
4	27.14	3	6 E-6	***
5	9.34	3	.025	*
6	7.81	4	.099	
7	19.34	4	7 E-4	***
8	62.96	4	7 E-13	***

*Note: * p<.05, ** p<.01 , *** p<.001 for the discrepancy between an observed and an expected distribution*

Conclusions

These analyses showed that the B-E model fit reasonably well the distributions of the CSVI data. Out of 21 whole-sample distributions, only 8 were significantly different from the model's predictions, often with only minor discrepancies; overall, there were just slight trends for the model to underestimate performance on the lowest levels and overestimate it on the highest ones (trends found in some but not all experiments). Out of 112 distributions from subsamples with a specific *k* score, only 9 were significantly different from the model's predictions.

In contrast, a binomial model based on some reasonable assumptions, but not including a limited capacity or specific attentional processes, clearly failed to account for the data.
