# Supplementary material for: Modelling Working Memory Capacity: Is the Magical Number Four, Seven, or Does it Depend on What You Are Counting?
Source: J Cogn. 2024 Jul 18;7(1):60. doi: 10.5334/joc.387 (PMC11259112; doi:10.5334/joc.387)
Supplement: Supplementary file 2. — Responses to single CSVI features. [file joc-7-1-387-s2.pdf]

S. Morra, P. Patella, L. Muscella - *Modelling Working Memory Capacity: Is the Magical Number Four, Seven, or Does it Depend on what you are Counting?*

Supplementary file 2

Responses to single CSVI features

The results reported in the main text of the article and in Supplementary file 1 provide considerable support for the Bose-Einstein model of CSVI performance. This Supplementary file 2 is devoted to detailed analyses of participants' responses to each feature of the stimuli, in order to explore whether they provide any indication of important assumption violations or possible alternative interpretations of the main results. Some of the issues considered here were already addressed by Pascual-Leone (1970, 1978), others have not yet been examined in the literature.

In particular, Pascual-Leone evaluated possible violations of the Bose-Einstein model's assumption that the *n* distinguishable states (in metaphor, the *n* distinguishable boxes; in the reality of the CSVI task, the nine relevant features of the stimuli) are equiprobable. As Pascual-Leone (1970, p.322) phrased it, the Bose-Einstein model implies the assumption that "they are equally salient and equally SR compatible with their respective responses". In case this assumption was violated, would the CSVI model be robust to that violation? In fact, Pascual-Leone (1970) found systematic differences in difficulty among the features used in that experiment (see in particular his Figure 1), which were consistent across age groups, and noted that this unevenness might cause a shift of the actual distributions of responses to some extent to the left with respect to those predicted by the model. However, violation of the assumption that all distinguishable states are equiprobable did not cause a major discrepancy between the observed and the expected distributions: "despite the noise introduced by the violation of assumptions, the theoretical predictions appear to be good for all ages and in particular for 9- and 11-year-olds" (Pascual-Leone, 1970, p. 327). Pascual-Leone (1978) discussed further this issue, stressing that feature equiprobability is not a prediction of his Theory of Constructive Operators but only a simplifying assumption necessary for testing the model, similar to other simplifying assumptions that we often make to analyze data or test models. Pascual-Leone (1978) indeed argued that the stimuli "must necessarily exhibit differences in salience due to differential overlearning of cues and/or Gestalt-like field factors […] The fact of unequal saliences is explicitly acknowledged […] and treated as simplifying assumption in the CSVI model. This effect of unequal salience of the different cues was also empirically evaluated […] The evaluation in fact suggested that salience differences have little effect on the developmental pattern of performances." (Pascual-Leone, 1978, p. 24).

Although Pascual-Leone (1970, 1978) already discussed extensively this issue, it should be noted that our experiments differ from those studies of the CSVI in several respects. His early research tested children of various ages, requiring different motor gestures as responses to stimulus features, and used a reduced set of features with younger children. In contrast, our experiments were carried out with adult or adolescent participants, with a fixed set of nine features and a computerized task, in which the required responses were presses of different buttons on a special keyboard. Moreover, in our experiments 1a-1c we also compared the traditional 5-sec presentation of the CSVI stimuli to three different brief presentation conditions. Thus, it seems advisable to check these experiments for possible violation of the equiprobability assumption, also as a function of presentation conditions, and consider whether any discrepancies from equiprobability might be indicative of particular strategies used by the participants to perform this task.

Experiments 1a-1c – correct responses

A preliminary analysis revealed that the order of conditions did not affect significantly the probability of detecting a feature and did not interact with experiments, conditions, and features. Therefore, it was not analyzed further. A mixed-design ANOVA with Experiment (3) as a between-subjects factor and Condition (2) and Feature (9) as within-subjects factors was carried out. The relevant means and standard deviations are reported in Table S2-1. All factors and their interactions were significant: Condition, $F(1, 57) = 209.13$, $p<.001$, $\eta_p = .786$; Feature, $F(8, 456) = 26.52$, $p<.001$, $\eta_p = .318$; Experiment, $F(2, 57) = 9.50$, $p<.001$, $\eta_p = .250$; Condition x Experiment, $F(2, 57) = 26.98$, $p<.001$, $\eta_p = .484$; Condition x Feature, $F(8, 456) = 31.00$, $p<.001$, $\eta_p = .352$; Feature x Experiment, $F(16, 456) = 5.68$, $p<.001$, $\eta_p = .166$; and Feature x Condition x Experiment, $F(16, 456) = 4.37$, $p<.001$, $\eta_p = .133$.

Table S2-1. Probability of correct response to each particular feature.

Feature	Condition							
	Long		Short Exp. 1a		Short Exp. 1b		Short Exp. 1c	
	Mean	(s.d.)	Mean	(s.d.)	Mean	(s.d.)	Mean	(s.d.)
Large size	.78	(.18)	.74	(.21)	.63	(.28)	.83	(.14)
Purple background	.81	(.21)	.88	(.13)	.31	(.31)	.64	(.36)
Frame	.84	(.18)	.67	(.20)	.71	(.20)	.83	(.18)
Dashed contour	.90	(.09)	.61	(.24)	.24	(.17)	.44	(.19)
Bar underneath	.91	(.10)	.77	(.17)	.50	(.37)	.82	(.22)
Square	.91	(.10)	.86	(.20)	.94	(.07)	.97	(.05)
Red	.92	(.09)	.85	(.14)	.89	(.09)	.95	(.06)
Circle in centre	.95	(.09)	.66	(.21)	.51	(.26)	.79	(.18)
X in center	.97	(.07)	.71	(.21)	.63	(.35)	.88	(.19)

Overall, the mean probability of responding correctly to a feature was .89 in the long and .71 in the short condition.

To clarify the interactions, separate analyses were carried out for the long and the short condition. In the ***long condition***, only the Feature factor was significant, $F(8, 456) = 14.73$, $p<.001$, $\eta_p = .205$. Neither the Experiment factor nor the interaction were significant, $F(2, 57) = 0.56$, $p>.47$, $\eta_p = .026$, and $F(16, 456) = 1.34$, $p>.16$, $\eta_p = .045$, respectively; this outcome may be unsurprising, because the long condition was identical in all three experiments.

To understand the pattern of different means for the single features, revealed by the significant feature factor, Bonferroni-corrected *t*-tests were carried out with a threshold probability of .05/36 = .0014. Large

size, purple background, and frame were the features least responded to, and their means did not differ significantly from one another. Red, circle in center, and X in center were the most probably detected features and their means did not differ significantly from one another. In the intermediate area, the dashed contour had a higher mean than large size and a lower mean than circle in center and X in center; the bar underneath had a higher mean than large size and a lower mean than X in center; the square shape had a higher mean than large size and purple background and a lower mean than X in center. It seems that the bright color or the central position in the figure made some features more salient perceptually than others.

In the ***short condition***, all effects were significant, namely, Feature, $F(8, 456) = 34.36$, $p<.001$, $\eta_p = .376$; Experiment, $F(2, 57) = 17.37$, $p<.001$, $\eta_p = .379$; and the interaction, $F(16, 456) = 6.83$, $p<.001$, $\eta_p = .193$. These findings reflect the different structure of the short condition in each experiment.

To understand how each kind of short condition altered the pattern of feature saliency with respect to the "classical" 5-sec presentation of the CSVI, we first considered the average difference between the long and short condition of each experiment. In experiment 1a, the probability of correct response to a feature decreased on average by .147 in the short with respect to the long condition; in experiment 1b it decreased by .294; and in experiment 1c, it decreased by .083.

Considering experiment 1a, the decrease in correct responses to each feature was compared with the value of .147, with the purpose of identifying which features suffered from the short condition more or less than the average for that experiment. These comparisons were carried out with Bonferroni-corrected one-sample *t*-tests with a threshold probability of .05/9 = .0056. The results indicated that the decrease of correct responses in the short condition was negligible for the purple background, $t(19) = -3.87$, $p=.001$, and for the red color, $t(19) = -3.52$, $p=.002$, whereas the circle in center decreased significantly more than the average of all features, $t(19) = 3.48$, $p=.003$. Also the dashed contour had a qualitatively notable decrease in the short condition, but with Bonferroni correction this was not significantly larger than average, $t(19) = 2.59$, $p=.018$. It seems that colors emerged almost as easily with a short presentation as with a long one, while some small, colorless features were less easily noted with a brief presentation.

Similarly, considering experiment 1b, the decrease in correct responses to each feature was compared with .294. Responses to the square shape and the red color did not suffer a decrease in the short condition, $t(19) = -15.59$, $p<.001$ and $t(19) = -10.02$, $p<.001$; also large size came close to significance for a smaller-than-average decrease, $t(19) = -3.06$, $p=.006$. In contrast, a very large drop in responses was found for the dashed contour, $t(19) = 10.80$, $p<.001$; the purple background and the circle in center were close to significance for a rather large drop, $t(19) = 2.90$, $p=.009$ and $t(19) = 2.69$, $p=.014$, respectively. In experiment 1b stimulus presentation was not only shorter than in experiment 1a, but also followed by a mask. It seems that only the macro-features of the main figure resisted very brief presentation and masking. Interestingly, the purple background that in the short condition of experiment 1a proved to be quite salient, in this experiment instead was effectively concealed by masks with backgrounds in bright colors.

Finally, considering experiment 1c, the decrease in correct responses to each feature was compared with the average value of .083. Similarly to experiment 1b, the features that remained salient notwithstanding (triple) brief, masked presentation were square shape, red color, and large size, $t(19) = -7.33$, $p<.001$, $t(19) = -6.12$, $p<.001$, and $t(19) = -4.40$, $p<.001$, respectively. In contrast, a large drop in responses was observed for the dashed contour, $t(19) = 8.74$, $p<.001$. Although triple presentation in this experiment yielded a higher rate of correct responses than in experiment 1b, the pattern across features was essentially the same. It seems likely that the different pattern observed in the short conditions of experiments 1a, 1b, and 1c was due to the unmasked presentation in experiment 1a versus masked presentation in experiments 1b and 1c.

In conclusion, it seems that the different probabilities of correct responses to features depended essentially on their perceptual salience or discriminability, and on the impact that long or short, masked or

unmasked presentation had on these perceptual aspects. We noted no hint that such differences might be a consequence of particular cognitive strategies of the participants. Similarly to Pascual-Leone (1970), we found that the assumption of equal probability of all distinguishable states was not realistic, but the Bose-Einstein model was quite robust to this assumption violation; indeed, as shown in Supplementary file 1, the Bose-Einstein distribution fit the data well. We suggest, as a possible reason why the Bose-Einstein model is robust to violation of this assumption, that (using the metaphor of boxes and balls) a box could contain one or more balls, but this is unimportant because the "particles" or "balls" are undistinguishable; thus, a single ball is sufficient to count the box as full. Out of metaphor, in the CSVI, more salient features probably tend to attract more "balls" (units of attention) than less salient features, but a single unit of attentional capacity allocated to a feature is sufficient for that feature to be consciously detected; therefore, unequal distribution of "balls" across "boxes" has only a minor impact on the number of correct responses.

Experiments 1a-1c – false alarms

We also analyzed false alarms, i.e., responses to features that were not actually present in a stimulus. In general, false alarms occurred rarely. A mixed-design ANOVA with Experiment (3) and Order of conditions (2) as between-subjects factors and Condition (2) as a within-subjects factor was carried out. A significant effect was found only for Condition, $F(1, 54) = 20.03$, $p<.001$, $\eta_p = .271$. All other effects were nonsignificant: Experiment, $F(2, 54) = 0.35$, $p>.71$, $\eta_p = .013$; Order, $F(1, 54) = 0.08$, $p>.78$, $\eta_p = .001$; Experiment x Order, $F(2, 54) = 0.59$, $p>.56$, $\eta_p = .021$; Experiment x Condition, $F(2, 54) = 0.32$, $p>.72$, $\eta_p = .012$; Order x Condition, $F(1, 54) = 0.33$, $p>.56$, $\eta_p = .006$; Experiment x Order x Condition, $F(2, 54) = 1.57$, $p>.21$, $\eta_p = .055$.

The mean probability of responding to a feature when it was not present was 0.016 in the long and 0.042 in the short condition. Comparing the probability of false alarms with hits (.89 in the long and, on average, .71 in the short condition) suggests that, with the "classical" long presentation, false alarms in the CSVI were really negligible. With short presentation false alarms were also rare, but not so rare as in the short condition, possibly indicating a slight tendency to occasional guessing when the stimuli were presented very briefly. As reported in the main text of this article, the difference between *k* estimates in the short and long conditions was nonsignificant in all experiments 1a-1c; however, there was consistently an unexpected, small (nonsignificant) difference of a few decimals in favor of the short condition. This could possibly be accounted for by the slight tendency to guess in the short condition – without which, the *k* estimates in both conditions would likely have been even closer to each other.

Experiments 1a-1c – order of responses

So far, to the best of our knowledge, the order of participants' responses to the different features of CSVI stimuli has never been examined. For instance, upon presentation of a level-*n* stimulus, a participant could give a maximum of *n* correct responses. In case a participant gives *x* responses to a stimulus, these could be produced in *x*! different orderings. The order of responses does not affect the scoring; however, it could be informative in other ways. Do participants produce their responses in a random order, or is the ordering affected by perceptual salience, or do participants have a preferred ordering of features for responding? In the latter case, this could be due either to factors of little cognitive relevance (e.g., following a simple path through the buttons on the keyboard) or to sophisticated cognitive strategies (e.g., rehearsing all features in a fixed order and checking whether each of them is present in a stimulus).

To explore the participants' ordering of responses, we coded for each participant all of the items on which he/she gave at least six responses. This was intended to ensure that response sequences were long enough for a meaningful analysis of their ordering. Thus, we considered in all 749 response sequences, of which 233 in the short and 516 in the long condition. We did not discard false alarms, because for the sake of ordering it seems of little relevance whether a participant actually saw a feature, or only believed or guessed having

seen it. The 749 sequences that we coded comprised in all 4880 responses, of which 97.9% were correct responses and 2.1% false alarms. Unfortunately, it was not possible to analyze the ordering separately in the long and short conditions, because out of 60 participants there were 14 who did not give at least six responses to any item in the short condition, and another 22 participants whose coded sequences in the short condition never included one or more of the nine features. Therefore, we proceeded analyzing together the response sequences produced in both conditions.

Each sequence was scored assigning an order rank to each feature; for instance, if on a certain item a participant pressed (in this order) the buttons for dashed contour, large, square, red, X in center, bar underneath, we assigned the scores 1 to dashed contour, 2 to large, and so on, ending with 6 to bar underneath. Then, having scored in this way all the relevant responses of a participant, we calculated the average score of that participant on each feature. For instance, if the feature frame appeared in nine of a participant's sequences, respectively in positions 4, 6, 2, 4, 3, 5, 7, 3, and 5, the average of these numbers would be 4.333; so, that participant would obtain a score of 4.333 for the feature frame – and similarly for all other features. Two participants had one missing feature in their set of coded sequences; in these two cases, the missing feature was imputed the same score as that participant's feature with the highest score (i.e., the one that tended to be responded to latest).

A mixed-design ANOVA with Feature (9) as a within-subject factor and Experiment (3) and Order of conditions (2) as between-subject factors yielded a significant result only for Feature, $F(8, 432) = 18.14$, $p<.001$, $\eta_p = .251$. The effects of Experiment, $F(2, 54) = 0.39$, $p>.67$, $\eta_p = .014$, Order of conditions, $F(1, 54) = 0.92$, $p>.34$, $\eta_p = .017$, and Experiment x Order of conditions, $F(2, 54) = 0.02$, $p>.98$, $\eta_p = .001$, were nonsignificant, as well as their interactions with Feature, i.e., Feature x Experiment, $F(16, 432) = 1.20$, $p>.26$, $\eta_p = .042$, Feature x Order of conditions, $F(8, 432) = 0.87$, $p>.54$, $\eta_p = .016$, and Feature x Experiment x Order of conditions, $F(16, 432) = 0.65$, $p>.84$, $\eta_p = .023$.

Table S2-2. Mean (and standard deviation) of each feature's positional rank.

Feature	Both conditions, including false alarms		Only long condition, excluding false alarms	
	Mean	(s.d.)	Mean	(s.d.)
Square	2.88	(1.01)	3.02	(1.16)
Circle in center	3.36	(1.01)	3.32	(1.09)
Red	3.48	(1.10)	3.41	(1.18)
Large size	3.55	(1.06)	3.68	(1.16)
X in center	3.56	(1.00)	3.55	(1.08)
Dashed contour	4.00	(0.80)	3.89	(0.89)
Purple background	4.17	(1.51)	4.30	(1.66)
Bar underneath	4.62	(0.96)	4.67	(1.12)
Frame	4.71	(0.79)	4.72	(0.92)

The means and standard deviations for each feature are reported in Table S2-2. To understand the pattern of different means for the single features, revealed by the significant feature factor, Bonferroni-corrected *t*-tests were carried out with a threshold probability of .05/36 = .0014. The square shape tended to be responded to earlier than other features; the Bonferroni-corrected *t*-tests were significant for comparison of square shape with every other feature except circle in center and X in center. In contrast, bar underneath and frame tended to be responded to latest; their orderings were not significantly different, and they were significantly different from all other features except the purple background. Furthermore, the circle in center was responded to significantly earlier than the dashed contour.

However, the analysis just reported might be biased or noisy, especially because sequences of at least six responses were particularly rare in the short condition of experiment 1b (i.e., there were 30 in all, with exactly half of the participants having no sequence of at least six responses). Also false alarms, albeit rare, might have introduced some undue noise. We repeated the analysis using a narrower but probably cleaner set of sequences, i.e., only those found in the long condition; we also dropped from this analysis the scores of the few false alarms. The descriptive statistics of this re-analysis are also reported in Table S2-2.

Also in this analysis, the only significant factor was Feature, $F(8, 432) = 14.49$, $p<.001$, $\eta_p = .212$. The other factors, i.e., Experiment and Order of conditions, and all interactions were nonsignificant, all with $F < 1$ and $p>.50$. Bonferroni-corrected *t*-tests revealed that square, circle in center, red, and X in center (i.e., the four features that were responded to earliest) did not differ significantly from one another. In contrast, bar underneath and frame tended to be responded to latest; their orderings were not significantly different, and they were significantly different from all other features except the purple background. Furthermore, square was responded to significantly earlier than large size, dashed contour, and purple background.

In sum, the findings for the temporal order of responses replicated to some extent (but not completely) those for probability of correct responses, because in general the perceptually most salient features that elicited with higher probability correct responses also tended to be responded to earlier. An effect of perceptual salience on the order of responses is quite consistent with the Bose-Einstein model of the CSVI, because perceptually salient features would likely attract more attentional units, and thus their schemes would be hyper-activated. There were exceptions, however. The square shape, that in the long condition had an intermediate probability of correct response, tended to be responded to earliest. We speculate that this could be due to a subjective sense of confidence; in some sense, the shape defines the “identity” of a figure, so, when a square shape was detected, several participants tended to respond to it first or second. The purple background had a somewhat larger standard deviation; although most participants tended to give delayed responses to it, some instead tended to respond to it early. The spatial arrangement of buttons on the keyboard could also have contributed to the ordering of responses. The button for large size was contiguous to that for square shape; thus, this spatial contiguity could have contributed to anticipated responses to large size. Similarly, bar underneath and frame not only did not have particularly high probability of correct responses, but also, the buttons associated with them were one near the other on the right side of the keyboard.

At the group level, the differences among features in temporal ordering of responses may be accounted for by a combination of perceptual salience, subjective confidence, and spatial proximity of the buttons, which do not seem to be the flags of a strategic ordering. However, these analyses at the group level could obscure individual differences in the ordering of responses, because different participants might have different preferred orders of features, and an analysis of group means could not reveal this. Informal examination of the response protocols suggests that only few participants seemed to have a preferred order of responses. A rather frequent observation, instead, was that when two or three items shared a sizable subset of (detected) features, the participant tended to repeat their ordering over a short series of items; this could be regarded as a form of priming. However, as soon as subsequent stimuli comprised different

features, that ordering of responses was disrupted and possibly replaced by a different one. For a more formal examination of possible individual differences, we can resort to indices of within-subject variability.

A first, simple index is the participant’s range of feature order scores. Participants who produced their responses in a completely random order would obtain similar order scores for the various features, and therefore, the range of these scores would be rather small. On the contrary, participants who followed a regular ordering of responses to the various features would have very low scores for some features and very high scores for other ones, and therefore a large range. Intermediate ranges would reflect intermediate situations, for instance, a participant prioritizing one or two features and responding in random order to the others, or a participant who was rather prone to the priming phenomenon described above, i.e., keeping a similar order of responses in a series of items that shared a sizable subset of features and then abandoning that order.

Figure S2-1. Frequency distribution of individual participants’ range of the features’ ordering scores. Left panel: both long and short conditions, false alarms included. Right panel: long condition only, false alarms excluded.

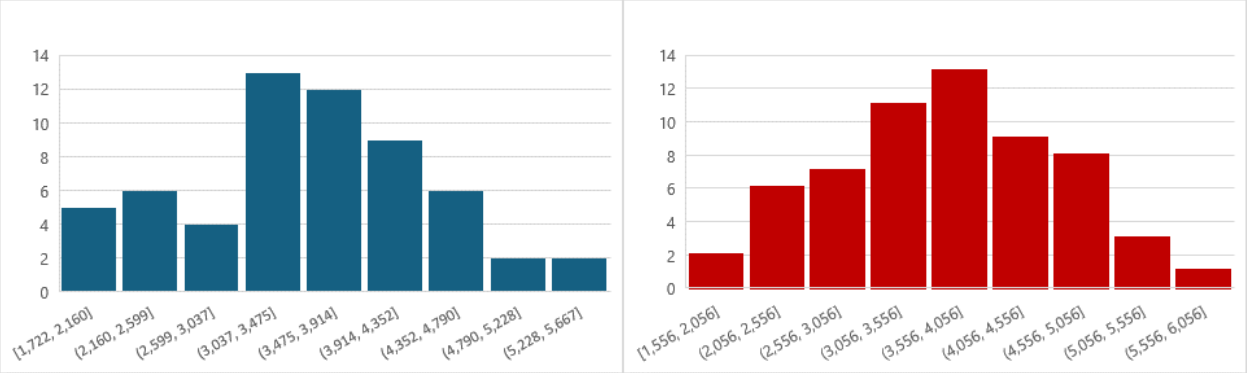

Figure S2-1 presents the distribution of such ranges. As one can see, the participants who responded in completely random order were very few. On the other hand, also the participants who responded quite consistently with a favorite order were very few. Consistent order of responses (if not merely due to a convenient path on the keyboard) could be an indicator of a clever strategy, based on sequential rehearsal and checking of a list of the features. As noted in the main text of the article, this strategy requires in the first place a very large working memory capacity for assembling it in the first place; perhaps for this reason, it is rare in young, well-educated adults and non-existing in younger populations. On the whole, one can regard the distribution reported in Figure S2-1 as showing that most participants were affected (perhaps some more and others less strongly affected) by the biases found at the group level, such as priority to the most salient features or consecutive responses to contiguous buttons; in addition, individual participants could also have idiosyncratic biases, such as a high priority to one or two particular features or a greater sensitivity to motor priming throughout some series of items.

Another, perhaps more reliable index is the participant’s standard deviation of feature scores. For the same reasons given for the range, participants responding in a random order would show small standard deviations of feature order scores, whereas participants following a consistent order would show large standard deviations.

Figure S2-2 presents the distribution of those standard deviations. It seems that the observations made to the distribution of individual participants’ ranges hold true also in the case of standard deviations.

Figure S2-2. Frequency distribution of individual participants' standard deviation of the features' ordering scores. Left panel: both long and short conditions, false alarms included. Right panel: long condition only, false alarms excluded.

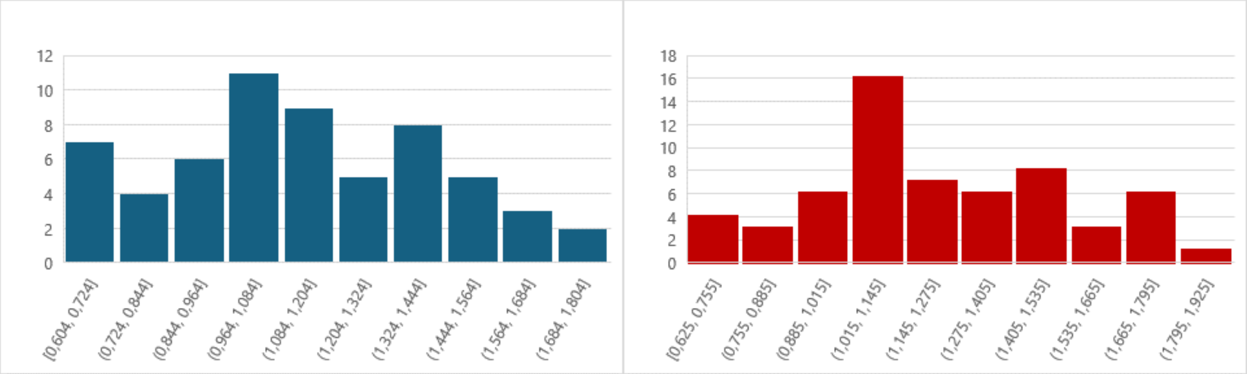

Finally, we submitted the feature order scores to exploratory factor analysis (principal components with varimax rotation). A preliminary analysis ensured that all variables approximated well normal distributions, with low values of skewness and kurtosis. Table S2-3 presents the results of the factor analyses, carried out using either the scores for both conditions pooled together or those of the long condition alone. The results were consistent in both analyses, with three factors accounting for 70.0% variance in the former analysis and 70.3% in the latter.

Table S2-3. Factor loadings of the feature order scores (only loadings ≥.40 in absolute value are reported).

Feature	Both conditions, including false alarms			Only long condition, excluding false alarms		
	F1	F2	F3	F1	F2	F3
Red	.70			.66		
Square	.62	-.52		.57	-.57	
Large size	.40	-.61		.53	-.48	
Dashed contour		.78			.81	
Frame		.75			.81	
Circle in center	-.83			-.85		
X in center	-.89			-.88		
Purple background			.90			.86
Bar underneath			.69			.78
% of total variance	29.3	21.7	19.0	29.6	21.2	19.5

The three factors that emerged from these analyses seem to reflect clearly distinct dimensions of attention allocation in the perceptual analysis of stimuli. The first factor denotes an opposition between macro-features of the figure and small details at the center. The second factor denotes a similar opposition between macro-features of the figure and peripheral details. The third factor refers to elements that surround the main figure.

The finding of these dimensions of perceptual-analysis-and-response is clearly interpretable in the light of Pascual-Leone's (1970) model of CSVI performance, which posits a number of distinct attending acts (e.g., *k* attending acts with the classical 5-sec presentation, or 3 attending acts in the short condition of our experiment 1c). It would seem plausible and convenient that a participant, while attending repeatedly to a stimulus, focuses his/her attention in turn on different areas; i.e., throughout a sequence of attending acts, attention also shifts in space. Still, each attending act is posited to be constrained by limited attentional capacity (*k* units) and random allocation of the available units of attentional capacity to the target features (possibly, the target features in a specific area of the stimulus). Future research on the CSVI could pursue further this finding by using eye tracking, and try to relate eye movements, order and timing of responses, and allocation of limited attentional capacity, in order to explore further the cycles of attending acts.

References

Pascual-Leone J. (1970). A mathematical model for the transition rule in Piaget's developmental stages. *Acta Psychologica, 32*, 301-345. doi: 10.1016/0001-6918(70)90108-3

Pascual-Leone J. (1978). Compounds, confounds and models in developmental information-processing: A reply to Trabasso and Foellinger. *Journal of Experimental Child Psychology, 26*, 18-40. doi: 10.1016/0022-0965(78)90106-6
